# Mn‐Rich MnSb_2_Te_4_: A Topological Insulator with Magnetic Gap Closing at High Curie Temperatures of 45–50 K

**DOI:** 10.1002/adma.202102935

**Published:** 2021-09-01

**Authors:** Stefan Wimmer, Jaime Sánchez‐Barriga, Philipp Küppers, Andreas Ney, Enrico Schierle, Friedrich Freyse, Ondrej Caha, Jan Michalička, Marcus Liebmann, Daniel Primetzhofer, Martin Hoffman, Arthur Ernst, Mikhail M. Otrokov, Gustav Bihlmayer, Eugen Weschke, Bella Lake, Evgueni V. Chulkov, Markus Morgenstern, Günther Bauer, Gunther Springholz, Oliver Rader

**Affiliations:** ^1^ Institut für Halbleiter‐ und Festkörperphysik Johannes Kepler Universität Altenberger Straße 69 Linz 4040 Austria; ^2^ Helmholtz‐Zentrum Berlin für Materialien und Energie Albert‐Einstein‐Straße 15 12489 Berlin Germany; ^3^ II. Institute of Physics B and JARA‐FIT RWTH Aachen Unversity 52074 Aachen Germany; ^4^ Institut für Physik und Astronomie Universität Potsdam Karl‐Liebknecht‐Straße 24/25 14476 Potsdam Germany; ^5^ Department of Condensed Matter Physics Masaryk University Kotlářská 267/2 Brno 61137 Czech Republic; ^6^ Central European Institute of Technology Brno University of Technology Purkyňova 123 Brno 612 00 Czech Republic; ^7^ Department of Physics and Astronomy Universitet Uppsala Lägerhyddsvägen 1 Uppsala 75120 Sweden; ^8^ Institute for Theoretical Physics Johannes Kepler Universität Altenberger Straße 69 Linz 4040 Austria; ^9^ Max Planck Institute of Microstructure Physics Weinberg 2 06120 Halle Germany; ^10^ Centro de Física de Materiales (CFM‐MPC) Centro Mixto CSIC‐UPV/EHU San Sebastián/Donostia 20018 Spain; ^11^ IKERBASQUE Basque Foundation for Science Bilbao 48011 Spain; ^12^ Peter Grünberg Institut and Institute for Advanced Simulation Forschungszentrum Jülich and JARA 52425 Jülich Germany; ^13^ Donostia International Physics Center (DIPC) San Sebastián/Donostia 20018 Spain; ^14^ Departamento de Polímeros y Materiales Avanzados: Física, Química y Tecnología, Facultad de Ciencias Químicas Universidad del País Vasco UPV/EHU San Sebastián/Donostia 20080 Spain; ^15^ Saint Petersburg State University Saint Petersburg 198504 Russia; ^16^ Tomsk State University Tomsk 634050 Russia

**Keywords:** magnetic bandgap, magnetic topological insulators, magnetization, MnSb _2_Te _4_, Mn–Sb site exchange, molecular beam epitaxy

## Abstract

Ferromagnetic topological insulators exhibit the quantum anomalous Hall effect, which is potentially useful for high‐precision metrology, edge channel spintronics, and topological qubits.  The stable 2+ state of Mn enables intrinsic magnetic topological insulators. MnBi_2_Te_4_ is, however, antiferromagnetic with 25 K Néel temperature and is strongly n‐doped. In this work, p‐type MnSb_2_Te_4_, previously considered topologically trivial, is shown to be a ferromagnetic topological insulator for a few percent Mn excess. i) Ferromagnetic hysteresis with record Curie temperature of 45–50 K, ii) out‐of‐plane magnetic anisotropy, iii) a 2D Dirac cone with the Dirac point close to the Fermi level, iv) out‐of‐plane spin polarization as revealed by photoelectron spectroscopy, and v) a magnetically induced bandgap closing at the Curie temperature, demonstrated by scanning tunneling spectroscopy (STS), are shown. Moreover, a critical exponent of the magnetization β ≈ 1 is found, indicating the vicinity of a quantum critical point. Ab initio calculations reveal that Mn–Sb site exchange provides the ferromagnetic interlayer coupling and the slight excess of Mn nearly doubles the Curie temperature. Remaining deviations from the ferromagnetic order open the inverted bulk bandgap and render MnSb_2_Te_4_ a robust topological insulator and new benchmark for magnetic topological insulators.

## Graphical abstract

Mn‐rich MnSb_2_Te_4_ is shown to exhibit ferromagnetic hysteresis with out‐of‐plane anisotropy and Curie temperature of 45–50 K. It is a topological insulator with Dirac point close to the Fermi level and a magnetic gap that closes at the Curie temperature. These favorable properties are caused by excess Mn that substitutes Sb within the septuple layers.

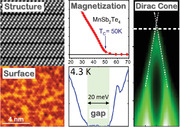

## 1 Introduction

The quantum anomalous Hall effect (QAHE) offers quantized conductance and lossless transport without the need for an external magnetic field.^[^ ^1^ ^]^ The idea to combine ferromagnetism with topological insulators for this purpose^[^ ^2^, ^3^, ^4^ ^]^ has fuelled the materials science.^[^ ^5^, ^6^ ^]^ It led to the experimental discovery of the QAHE in Cr‐ and V‐doped (Bi, Sb)_2_Te_3_^[^ ^7^, ^8^, ^9^, ^10^, ^11^ ^]^ with precise quantized values of the Hall resistivity down to the sub‐part‐per‐million level.^[^ ^12^, ^13^, ^14^, ^15^ ^]^ The stable 3+ configuration of V or Cr substitutes the isoelectronic Bi or Sb^[^ ^3^, ^16^, ^17^ ^]^ enabling ferromagnetism by coupling the magnetic moments of the transition metal atoms. Hence, time‐reversal symmetry is broken enabling ‐ through perpendicular magnetization ‐ a gap opening at the Dirac point of the topological surface state.^[^ ^2^, ^3^, ^4^, ^5^ ^]^ This gap hosts chiral edge states with precisely quantized conductivity. However, the experimental temperatures featuring the QAHE are between 30 mK^[^ ^7^, ^13^ ^]^ and a few K^[^ ^18^, ^19^ ^]^ only, significantly lower than the ferromagnetic transition temperatures *T*_C_ in these systems.^[^ ^20^ ^]^ If the temperature of the QAHE could be raised, applications such as chiral interconnects,^[^ ^21^ ^]^ edge state spintronics,^[^ ^22^, ^23^ ^]^ and metrological standards^[^ ^14^, ^15^ ^]^ become realistic.

One promising approach is the so‐called modulation doping in which the magnetic dopants are located only in certain parts of the topological insulator. This implies strong coupling of the topological surface state to the magnetic moments at a reduced disorder level.^[^ ^18^, ^24^ ^]^ Most elegantly, this has been realized for Mn‐doped Bi_2_Te_3_ and Bi_2_Se_3_. The tendency of Mn to substitute Bi is weak, such that Mn doping leads to the spontaneous formation of septuple layers with MnBi_2_Te_4_ stoichiometry. These septuple layers are statistically distributed among quintuple layers of pure Bi_2_Te_3_ or Bi_2_Se_3_ at low Mn concentration^[^ ^25^, ^26^ ^]^ and increase in number with increasing Mn concentration.^[^ ^25^ ^]^ Eventually, only septuple layers remain when the overall stoichiometry of MnBi_2_Te_4_ or MnBi_2_Se_4_^[^ ^26^, ^27^ ^]^ is reached. Density functional theory (DFT) calculations found that MnBi_2_Te_4_ forms ferromagnetic layers with antiferromagnetic interlayer coupling^[^ ^28^, ^29^ ^]^ as confirmed by experiments at low temperatures.^[^ ^29^, ^30^, ^31^, ^32^ ^]^ As a result, MnBi_2_Te_4_ is an antiferromagnetic topological insulator^[^ ^29^, ^33^, ^34^, ^35^, ^36^ ^]^ that can exhibit axion states.^[^ ^5^ ^]^ The QAHE, however, has only been realized in a limited way: ultra thin flakes consisting of odd numbers of septuple layers exhibited an anomalous Hall effect (AHE) that is nearly quantized. This is caused by the uncompensated ferromagnetic septuple layer without partner. Nevertheless, exact quantization still required a magnetic field.^[^ ^37^ ^]^ A ferromagnetic AHE has also been observed for systems with either a larger amount of quintuple layers^[^ ^38^, ^39^, ^40^ ^]^ or via alloying of Sb and Bi in MnBi_2−*x*_Sb_*x*_Te_4_^[^ ^31^, ^32^, ^41^ ^]^ or both.^[^ ^42^, ^43^ ^]^ Most notably, a nearly quantized AHE has been observed up to 7 K for a MnBi_2_Te_4_/Bi_2_Te_3_ heterostructure after unconventional counter doping inducing vacancies by electron bombardment.^[^ ^19^ ^]^

A central drawback of the Bi_2_Te_3_ and Bi_2_Se_3_ host materials is their strong n‐type doping. In contrast, Sb_2_Te_3_ is p‐doped and much closer to charge neutrality.^[^ ^44^ ^]^ Indeed, mixtures of Bi_2_Te_3_ and Sb_2_Te_3_ with stoichiometries close to Sb_2_Te_3_ have been employed for the QAHE using Cr and V doping.^[^ ^7^, ^8^, ^9^, ^10^, ^11^ ^]^ Magnetism of dilute Mn‐doped Sb_2_Te_3_ has initially been studied by Dyck *et al*. obtaining *T*_C_ ≃ 2 K and perpendicular anisotropy.^[^ ^45^ ^]^ Later, a higher *T*_C_ = 17 K was reported for 1.5% Mn doping.^[^ ^46^ ^]^ Stoichiometric bulk MnSb_2_Te_4_ provided antiferromagnetism (Néel temperature *T*_N_ = 20 K)^[^ ^31^, ^32^, ^47^ ^]^, ferromagnetism^[^ ^48^ ^]^ as well as ferrimagnetism^[^ ^47^, ^49^ ^]^ (*T*_C_ = 25–34 K), depending on the composition and synthesis conditions. By comparison with scattering methods, it has been conjectured that this is related to Mn–Sb site exchange within the septuple layers^[^ ^42^, ^47^, ^50^ ^]^ which could even lead to spin glass behaviour.^[^ ^51^ ^]^ DFT calculations found that the perfectly ordered MnSb_2_Te_4_ is antiferromagnetic^[^ ^28^ ^]^ but topologically trivial,^[^ ^47^, ^52^, ^53^, ^54^, ^55^ ^]^ while Mn–Sb site exchange can render the interlayer coupling ferromagnetic.^[^ ^47^, ^50^ ^]^

Here, epitaxial MnSb_2_Te_4_ is studied using spin‐ and angle‐resolved photoemission spectroscopy (ARPES), scanning tunneling microscopy (STM) and STS, magnetometry, X‐ray magnetic circular dichroism (XMCD) and DFT. All experiments were performed as a function of temperature to pin down the intricate correlation between magnetism and non‐trivial band topology essential for the QAHE. It is revealed that the material unites the favorable properties of a topological insulator with its Dirac point close to the Fermi level *E*_F_ with that of a ferromagnetic hysteresis with out‐of‐plane anisotropy and record‐high *T*_C_, twice as high as the *T*_N_ previously reported for antiferromagnetic MnBi_2_Te_4_ and MnSb_2_Te_4_.^[^ ^31^, ^32^ ^]^ Moreover, temperature dependent STS finds a magnetic gap of 17 meV at *E*_F_ for 4.3 K that closes exactly at *T*_C_ as expected for a ferromagnetic topological insulator. Moreover, by combining DFT, STM, Rutherford backscattering, and X‐ray diffraction (XRD) it is uncovered that a partial substitution of Sb atoms by Mn is decisive to render MnSb_2_Te_4_ both ferromagnetic and a topological insulator.

## 2 Structure

**Figure 1:**
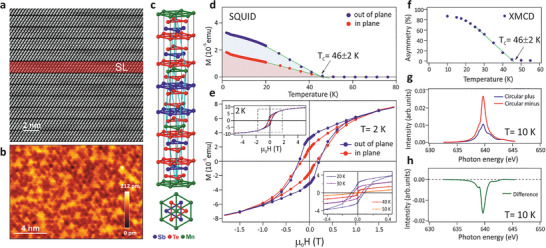
Structural and magnetic properties of epitaxial MnSb_2_Te_4_. a) Cross‐sectional HAADF‐STEM image of the septuple layer sequence formed in the MnSb_2_Te_4_ films on BaF_2_ substrates. For clarity, one of the septuple layers is highlighted by red color and denoted as SL. The weak van‐der‐Waals‐like bond between the Te‐terminated septuple layers is the natural surface termination. b) STM image of the flat topography of this surface, recorded at *T* = 4.3 K, sample voltage of 500 mV and current of 200 pA. c) Sketch of the ideal crystal structure as side view and top view. d) SQUID magnetometry as a function of temperature while cooling the sample in 10 mT from 300 K down to 2 K. e) Hysteresis loops probed by SQUID. The insets show a larger *H* range and the temperature dependence. Perpendicular anisotropy can be deduced from the larger out‐of‐plane signal. f) Temperature‐dependent XMCD signal at the Mn *L*_3_‐edge and the (0001) Bragg peak position recorded in X‐ray scattering geometry at 0 T after field cooling in 0.5 T. g) Corresponding full XMCD spectra recorded with opposite circular polarizations at 10 K. h) Difference of the spectra in (g). Linear extrapolation in (d,f) (green lines) reveals a ferromagnetic Curie temperature *T*_C_ = 46 ± 2 K for both experimental probes.

Epitaxial MnSb_2_Te_4_ films with 200 nm thickness were grown by molecular beam epitaxy (MBE) using an Mn:Sb_2_Te_3_ flux ratio of 1:1 in order to obtain the desired 1:2:4 stoichiometry of the MnSb_2_Te_4_ phase (Note S1, Supporting Information). **Figure** **1**a shows the cross section of the MnSb_2_Te_4_ lattice structure revealed by high‐resolution high‐angle annular dark‐field scanning transmission electron microscopy (HAADF‐STEM). It consists of septuple layers (SL) with stacking sequence Te–Sb–Te–Mn–Te–Sb–Te, Figure 1c, that corresponds to the MnSb_2_Te_4_ stoichiometry. The composition was verified by Rutherford backscattering spectrometry (RBS), indicating a small excess of Mn of 6% in the layers (composition of Mn_1.06_Sb_1.94_Te_4_, see Figure S1, Supporting Information), which is attributed to the fact that a small amount of Sb_2_Te_3_ desorbs from the surface during growth. Like with MnBi_2_Te_4_/Bi_2_Te_3_,^[^ ^56^ ^]^ the exchange coupling is strongly enhanced in these septuple layers relative to a disordered system where Mn substitutes Bi randomly.

The nearly exclusive formation of septuple layers in the entire MnSb_2_Te_4_ samples is confirmed by high‐resolution XRD (Figure S2, Supporting Information), revealing only a minute number of residual quintuple layers. In contrast, STEM and XRD of V‐doped (Bi, Sb)_2_Te_3_ shows quintuple layers only.^[^ ^57^ ^]^ This highlights that septuple layers are unfavorable for V^3+^ incorporation but very favorable for Mn as this requires the addition of a charge neutral transition metal^2+^/Te^2−^ bilayer to each quintuple layer, which is possible for Mn^2+^ but not for V^3+^. In addition, detailed XRD analysis reveals a 10% Mn–Sb site exchange within the septuple layers (see Figure S2, Supporting Information), meaning that Mn does not reside exclusively in the center of the septuple layers but also to a small extent on Sb sites in the adjacent cation layers. The amount of Mn–Sb site exchange is, however, significantly smaller as compared to single crystals reported to be of the order of 30–40%.^[^ ^47^, ^49^ ^]^ This is due to the much lower growth temperature of our epilayers of 290 °C compared to 600–650 °C for single crystals. Indeed, STM images of the atomically flat and Te‐terminated surface of our MnSb_2_Te_4_ epilayers (Figure 1b) exhibit triangular features, pointing to defects in the cation layer beneath the surface.^[^ ^58^, ^59^ ^]^ These defects occur with an atomic density of 5–10%. Since this is larger than for undoped Sb_2_Te_3_ films,^[^ ^58^ ^]^ the triangles are most likely caused by subsurface Mn atoms on Sb sites, in line with the XRD and RBS results. As shown by DFT below, these defects turn out to be decisive for the ferromagnetic interlayer coupling in MnSb_2_Te_4_. Similar conjectures have been raised previously.^[^ ^42^, ^47^, ^50^, ^51^, ^60^ ^]^

## 3 Magnetic Properties

Figure 1d displays the temperature‐dependent magnetization *M*(*T*) measured by superconducting quantum interference device (SQUID) magnetometry. The measurements were recorded while cooling the sample from 300 K to 2 K in a field of 10 mT perpendicular (blue) or parallel (red) to the film surface. Most strikingly, the MnSb_2_Te_4_ epilayers show pronounced ferromagnetic behavior by *M*(*H*) hysteresis loops (Figure 1e) with record high *T*_C_ of 45–50 K (Figure 1d), revealed independently for several samples (Figure S3, Supporting Information). This is significantly higher than the antiferromagnetic or ferromagnetic transition temperatures of bulk crystals (Table I, Supporting Information). Moreover, it is twice as large as the *T*_N_ ≤ 25 K of MnBi_2_Te_4_ films grown under the same conditions. It has been crosschecked that the displayed magnetization in *M*(*T*) curves is identical to the corresponding hysteresis loops. In particular, the large remanent magnetization observed by the bulk sensitive SQUID measurements excludes that it is caused by uncompensated antiferromagnetically coupled septuple layers only.^[^ ^37^ ^]^ However, the *M*(*H*) hysteresis curve (Figure 1e) shows a rounded shape that persists up to fields much higher than those typical for domain reversals and it does not saturate up to ±5 T where the average magnetic moment per Mn atom is of the order of 1–1.5μ_B_, similar to that of bulk MnSb_2_Te_4_ single crystals.^[^ ^31^, ^47^, ^49^ ^]^ This is because very high fields of 60 T are required to fully polarize the system^[^ ^61^ ^]^ and is in line with a similar rise of the Hall resistance with applied field in Figure S10, Supporting Information. This suggests additional types of competing magnetic orders. Indeed, a small kink in *M*(*T*) is observed at 20–25 K (Figure S3, Supporting Information), close to the Néel temperature reported earlier for antiferromagnetic MnSb_2_Te_4_.^[^ ^31^ ^]^ This implies that the high‐temperature ferromagnetism is most likely accompanied by ferrimagnetism as also supported by the relatively large in‐plane hysteresis and magnetization, Figure 1e, in line with observations of competing ferro‐ and antiferromagnetic order in bulk MnSb_2_Te_4_.^[^ ^40^, ^42^, ^47^, ^48^, ^50^, ^51^, ^61^ ^]^

Ferromagnetism is fully confirmed by element specific zero‐field XMCD recorded in diffraction geometry (see Note S7, Supporting Information). For these measurements, the sample was remanently magnetized at ≈0.5 T and 10 K. From spectra recorded with oppositely circularly polarized light at the (0001) Bragg peak, the intensity difference *C*_+_ − *C*_−_ (Figure 1h) was deduced for 0 T with the photon energy tuned to the Mn‐*L*_3_ resonance (Figure 1g). The asymmetry (*C*_+_ − *C*_−_)/(*C*_+_ + *C*_−_) directly yields the magnetization of the Mn atoms. Its temperature dependence (Figure 1f) impressively confirms the SQUID data with high *T*_C_ ≃ 46 K and unambiguously attributes the ferromagnetism to the Mn atoms. (For magnetotransport data, see Figure S10, Supporting Information.) The record‐high *T*_C_ with magnetic easy axis perpendicular to the surface (crystallographic *c*‐axis) as well as the large coercivity (≈0.2 T) render the samples very robust ferromagnets. Note that the spin‐orbit interaction is crucial for both out‐of‐plane easy axis and large coercivity.^[^ ^25^ ^]^ While it is sufficiently strong to turn the magnetization out of plane in Mn‐doped Bi_2_Te_3_, the effect in Mn‐doped Bi_2_Te_3_ is too weak.^[^ ^25^, ^62^ ^]^ The present data reveals that Sb_2_Te_3_ is sufficiently heavy, that is, spin‐orbit coupling sufficiently large, to maintain the perpendicular anisotropy for high Mn content.

To elucidate the origin of the ferromagnetism, DFT calculations are employed (Section S11, Supporting Information). They firstly highlight the differences between MnSb_2_Te_4_ and MnBi_2_Te_4_. In both cases, the in‐plane Mn coupling within each septuple layer is ferromagnetic. It is unlikely that the observed difference between antiferromagnetic interlayer coupling in MnBi_2_Te_4_ and ferromagnetic interlayer coupling in MnSb_2_Te_4_ is caused by the lowered spin‐orbit interaction, since it obviously remains large enough to induce a strong out‐of‐plane anisotropy in both cases. However, the in‐plane lattice constant *a* of MnSb_2_Te_4_ is ≈2% smaller than for MnBi_2_Te_4_. Hence, we calculated the exchange constants of MnSb_2_Te_4_ for *a* determined by XRD in comparison to those for *a* expanded to the value of MnBi_2_Te_4_ (see Table III, Supporting Information). Although the smaller in‐plane lattice constant of MnSb_2_Te_4_ increases the in‐plane exchange constant *J* between nearest neighbors by almost a factor of three, *T*_N_ remains barely changed because the enlarged in‐plane overlap of the Mn *d* states concomitantly weakens the already small perpendicular interlayer coupling. However, the energy gain of antiferromagnetism against ferromagnetism becomes as low as 0.6 meV per Mn atom at the XRD lattice constant. This suggests that already small structural changes along the interlayer exchange path can easily induce the transition to ferromagnetic order.

Indeed, a small degree of Mn–Sb site exchange occurs in the setpuple layers, meaning that a small fraction of Mn actually resides on Sb sites. Including this site exchange, our DFT calculations reveal that already 2.5% of Mn on Sb sites and, in turn, 5% of Sb on Mn sites is sufficient to swap the sign of the interlayer exchange constant (see Table III, Supporting Information). This renders MnSb_2_Te_4_ ferromagnetic for a degree of site exchange that agrees well with our XRD analysis and the density of subsurface defects observed by STM (Figure 1b). An even stronger site exchange has been recently reported for bulk MnSb_2_Te_4_^[^ ^47^, ^49^, ^51^ ^]^ and MnSb_1.8_Bi_0.2_Te_4_ single crystals,^[^ ^32^ ^]^ which corroborates that the Mn–Sb site exchange easily occurs. Thus, we conjecture that MnSb_2_Te_4_ single crystals remain antiferromagnetic^[^ ^32^ ^]^ only for negligible Sb–Mn intermixing. It is noted that Mn on Sb sites tends to couple antiferromagnetically to the Mn in the central layer of the septuple layer, while the Mn moments in the center of the septuple layers always couple ferromagnetically to each other.

According to our DFT calculations, however, Mn–Sb site exchange barely increases the transition temperature (*T*_N_ = 18 K → *T*_C_ = 25 K, see Table III, Supporting Information). This can only be achieved by incorporation of excess Mn in the DFT calculations, while keeping the Mn concentration in the central layer constant. In fact it turns out that already a small Mn excess of 5% residing on the Sb sites strongly increases *T*_C_ to 44 K, which well reproduces the experimental *T*_C_ = 45–50 K values. The strong enhancement is caused by the simultaneous strengthening of the intra‐ and interlayer exchange constants (see Table III, Supporting Information). The conclusion is robust toward charge doping by up to 0.2% Te or Sb vacancies that negligibly changes *T*_C_ (Note S11, Supporting Information). Note that the excess Mn in the Sb layers is perfectly in line with our RBS results (Figure S1, Supporting Information) indicating a Mn excess of 6% in our samples.

## 4 Topology and Magnetic Gap

**Figure 2:**
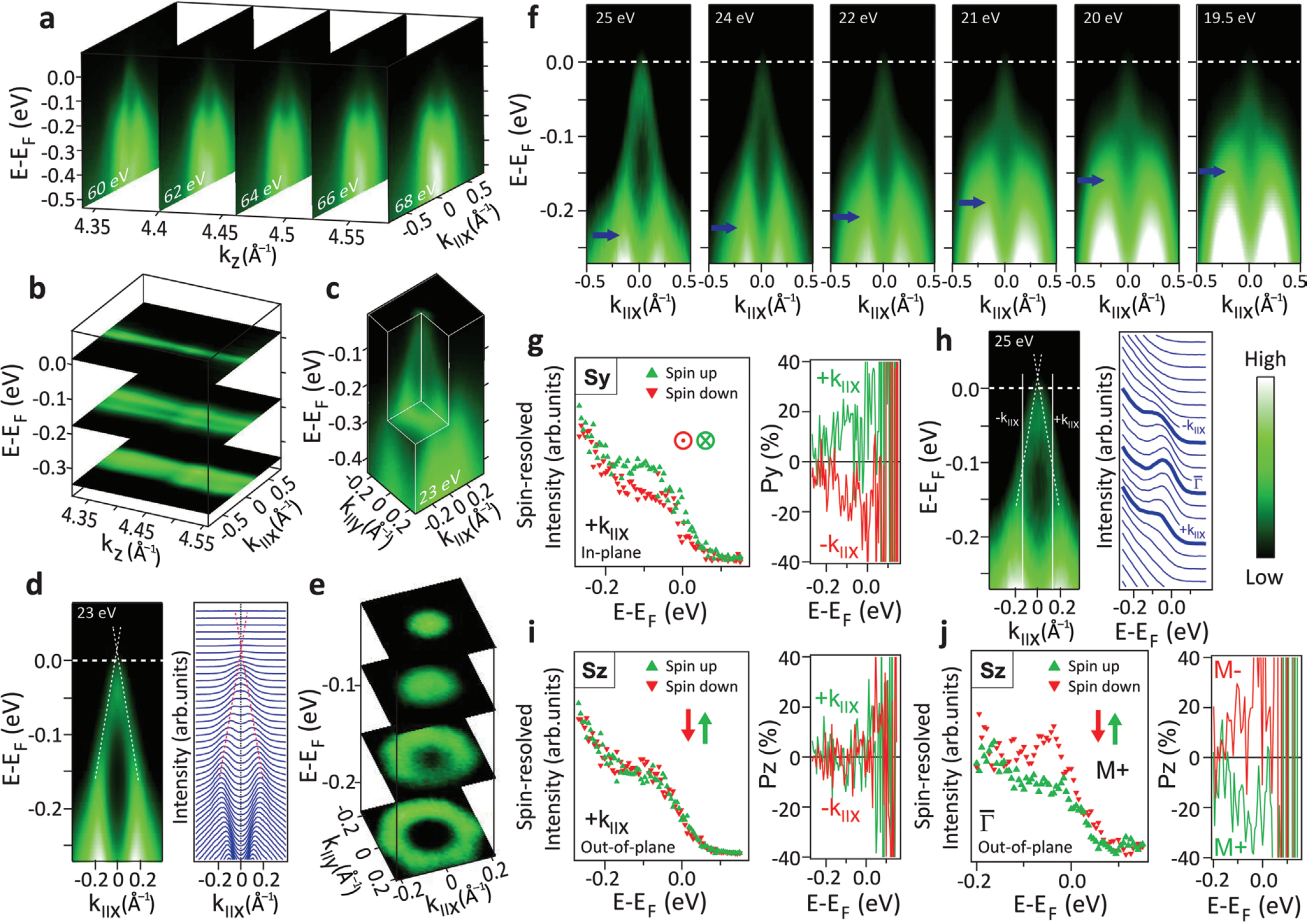
Topological properties of MnSb_2_Te_4_ revealed by ARPES. a) ARPES maps along *E*(*k*_∥*x*_) for five different photon energies *hν* displayed at the deduced *k*_*z*_ values. b) Same data as (a) displayed as (*k*_∥*x*_, *k*_*z*_) maps via interpolation featuring negligible dispersion along *k*_*z*_. The *k*_*z*_ range covers the whole Brillouin zone. c) Full 3D representation of the surface Dirac cone with *k*_∥*x*_ pointing along the $\bar{\Gamma }$ – $\bar{K}$ direction. d) Energy‐momentum dispersion of the Dirac cone (left) and corresponding momentum‐distribution curves (right) recorded at *hν* = 23 eV. The dashed, red lines along maxima result from fits to the data (Note S8, Supporting Information). They extrapolate to a Dirac point 20 meV above the Fermi level. e) Constant‐energy cuts of the Dirac cone. f) ARPES maps along *E*(*k*_∥,*x*_) at different photon energies featuring a strong dispersion of a bulk valence band (blue arrows) with photon energy (i.e., *k*_*z*_). g,i) Spin‐resolved ARPES at *k*_∥_ as marked in (h), hence, crossing the surface Dirac cone. Left: spectra for the two spin channels at one *k*_∥,*x*_. Right: spin polarization for both *k*_∥,*x*_, that is, ±*k*_∥,*x*_. g) In‐plane spin direction *S*_*y*_ perpendicular to *k*_∥_. i) Out‐of‐plane spin direction *S*_*z*_. Data for *S*_*x*_: Figure S4, Supporting Information. h) Left: surface Dirac cone with marked *k*_∥_ of the spectra in (g,i) and dashed line along intensity maxima as deduced by fitting (Note S8, Supporting Information). Right: energy distribution curves showing where (g,i,j) were measured. j) Spin‐resolved ARPES recorded at $\bar{\Gamma }$ and 30 K, and showcasing an out‐of‐plane spin polarization that reverses sign with reversal of the sample magnetization (M^+^ and M^−^, right).

Next, we probe the topological properties of the epitaxial MnSb_2_Te_4_ layers, recalling that perfectly stoichiometric MnSb_2_Te_4_ has been predicted to be topologically trivial.^[^ ^47^, ^52^, ^53^, ^54^ ^]^ In particular, it could be turned into a topological insulator only by replacement of more than half of the Sb by Bi^[^ ^53^ ^]^ or by compressing the lattice by as much as 3%.^[^ ^52^ ^]^ However, as shown by **Figure** **2**, our ARPES data from the MnSb_2_Te_4_ epilayers reveal the existence of a topological surface state with the dispersion of a Dirac cone along the wave vector parallel to the surface **k**_∥_. Varying the photon energy (Figure 2a,b and Figure S5, Supporting Information) to tune the electron wave number *k*_*z*_ perpendicular to the surface once through the whole bulk Brillouin zone (Table II, Supporting Information) reveals no dispersion, evidencing the 2D character of the Dirac cone, contrary to the lower‐lying 3D bulk bands that strongly disperse with photon energy (Figure 2f, arrows). Moreover, spin‐resolved ARPES of the 2D Dirac cone showcases a helical in‐plane spin texture at **k**_∥_ away from the $\bar{\Gamma }$ zone center, that is, it exhibits the characteristic reversal of spin orientation with the sign of **k**_∥_ (Figure 2g,i, Figure S4, Supporting Information). This spin chirality is a key signature of a topological surface state. In addition, a pronounced out‐of‐plane spin polarization of about 25% occurs in the remanently magnetized sample at the $\bar{\Gamma }$ zone center in the vicinity of *E*_F_, which reverses its sign when the sample is magnetized in the opposite direction (Figure 2j). Such out‐of‐plane spin texture at $\bar{\Gamma }$ is evidence for a magnetic gap opening at the Dirac point.^[^ ^63^ ^]^ Combined, the ARPES results demonstrate that MnSb_2_Te_4_ is a ferromagnetic topological insulator with clear fingerprints of a magnetic gap at $\bar{\Gamma }$. Favorably, the Dirac point of the topological surface state is rather close to *E*_F_. Indeed, extrapolation of the observed linear bands, deduced by Lorentzian peak fitting (Figure 2d,h), yields a position of the Dirac point *E*_D_ of 20 ± 7 meV above *E*_F_ at 300 K (Note S8, Supporting Information).

**Figure 3:**
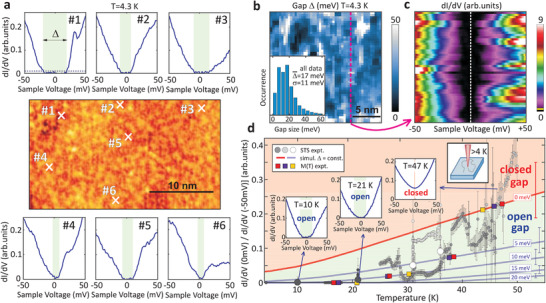
Spatial variation of the magnetic energy gap and its closing at *T*_C_. a) Center: STM image of MnSb_2_Te_4_ recorded at *T*= 4.3 K after ultrahigh vacuum sample transfer from the MBE system, *V* = 0.5 V, *I*= 0.2 nA. Surrounding: *dI*/*dV* spectra recorded at the crosses as labelled. The dashed line in the upper left spectrum is the noise threshold used for determination of gap size Δ (Note S9, Supporting Information). b) Spatial map of gap size Δ. Inset: histogram of Δ resulting from three distinct areas, average gap size $\bar{\Delta }$ and standard deviation σ are marked. c) d*I*/d*V* spectra (color code) along the magenta dashed line in (b). The yellow line at the bottom of the color code bar marks the threshold for gap determination in (a,b). d) Small dots: Ratio R=[dI/dV(V=0 mV)]/[dI/dV(V=−50 mV)] deduced from *dI*/*dV*(*V*) curves recorded at different *T*. The different gray shades correspond to different cooling cycles. Each point belongs to a single position on the sample surface within 1 nm. The error bars represent the standard deviation resulting from multiple curves measured at the same position. Large gray dots: average values of all data at corresponding *T* implying spatially averaged gap sizes $\bar{\Delta }$(21 K) = 11 ± 5 meV, $\bar{\Delta }$(31 K) = 6 ± 1 meV, and $\bar{\Delta }$(36 K) = 4 ± 1 meV (Note S9, Supporting Information). Gray lines: *T*‐dependence of the ratio *R* for the marked gap sizes $\bar{\Delta }$ as deduced by convolving the measured *dI*/*dV* data at 4.3 K with the Fermi distribution function (Figure S9b, Supporting Information). The area above Δ = 0 meV exhibits such a large *R* that the existence of a gap can be excluded. The error bars marked on the right result from the variation of the d*I*/d*V* curves at 4.3 K and represent a standard deviation (Figure S9b, Supporting Information). Colored squares: gap values deduced from XMCD (yellow, Figure 1f) and SQUID (red, violet, Figure S3, Supporting Information) via $\bar{\Delta }(T)\propto M(T)$.^[^ ^70^ ^]^ Insets: selected d*I*/d*V* curves belonging to the points with blue arrows. For additional curves see Figure S8, Supporting Information.

Since a Dirac point above *E*_F_ is not accessible for ARPES, here we employ low temperature STS to directly probe the ferromagnetic gap expected to open up at *T* < *T*_C_. **Figure** **3**a shows a topography STM image of the MnSb_2_Te_4_ epilayer at 4.3 K together with six STS spectra recorded at different locations on the surface. All spectra consistently reveal a gap at *E*_F_ varying, however, significantly in size. Attributing the energy region where d*I*/d*V*≈0 as gap (Note S9, Supporting Information), a full map of the gap size Δ is obtained for a larger surface region (Figure 3b),^[^ ^64^, ^65^ ^]^ with the corresponding gap histogram displayed as inset. The gap size varies in the range 0–40 meV with a mean value of 17 meV and a spatial correlation length of 2 nm observed consistently in three distinct areas (Figure S7, Supporting Information). A corresponding set of d*I*/d*V* curves recorded along the dashed line in Figure 3b is depicted in Figure 3c and showcases the small‐scale bandgap fluctuations. Likely, the spatial variation of Δ is caused by the spatially varying subsurface defect configurations, that is, by the Mn atoms on Sb lattice sites. Indeed, the gap fluctuations appear on the same length scale as the topographic features in Figure 1b. The average gap center position is only 0.6 meV above *E*_F_ (Figure S7, Supporting Information), which compares well to the value of *E*_D_ − *E*_F_ = 20 meV determined by ARPES on another sample. The small difference most likely arises from small differences in the growth and surface conditions, but may also be caused by the different measurement temperatures (4.3 K vs 300 K) or larger‐scale potential fluctuations.^[^ ^66^, ^67^ ^]^

To prove that the energy gap is of magnetic origin,^[^ ^25^ ^]^ the temperature dependence Δ(*T*) is probed by STS. This has not been accomplished yet for any magnetic topological insulator because at higher temperatures *k*_B_ *T* ≥ Δ/5 the STS gap Δ is smeared by the broadening of the Fermi–Dirac distribution, leading to a small non‐zero tunneling current at voltages within the bandgap.^[^ ^68^ ^]^ A direct deconvolution of the local density of states (LDOS) and the Fermi distribution function would require an assumption on the LDOS shape as function of energy. Such an assumption is impeded due to the significant spatial variation of the d*I*/d*V* curves at 4.3 K (Figure 3a–c), as also found consistently for other magnetic topological insulators.^[^ ^64^, ^69^ ^]^ Therefore, we developed a new method to derive Δ at elevated temperatures using the ratio between d*I*/d*V* at *V* = 0 mV and d*I*/d*V* at *V* well outside the region of the 4.3 K bandgap (Note S9, Supporting Information). Figure 3d displays the ratio *R* = [d*I*/d*V*(0 mV)]/[d*I*/d*V*(−50 mV)] for a large number of d*I*/d*V* spectra recorded at temperatures varying between 4.3 K and 50 K (small dots). Selected d*I*/d*V* spectra at 10, 21 and 47 K are shown as insets (more d*I*/d*V* data in Figure S8, Supporting Information).

One can see that up to 20 K, the STS ratio *R* is zero and thus d*I*/d*V* = 0 nS at zero bias, directly evidencing the persistence of the gap. At higher temperatures the *R* values gradually increase, which is due to the convolution of the temperature broadening effect along with the decreasing magnetic gap size that goes to zero at *T* = *T*_C_. To separate these two effects, we model the temperature dependence of the STS ratio for fixed gap sizes Δ(4.3K) = 0, 5, 10, 15 and 20 meV by convolving the d*I*/d*V* curves recorded at 4.3 K (Figure 3a–c) with the Fermi–Dirac distribution (Figure S9, Supporting Information). The corresponding model results are represented by solid gray lines in Figure 3d, where the red line marked with 0 meV indicates how the STS ratio *R* would evolve with temperature when the gap is zero. Clearly, up to *T* = 47 K, the experimental STS data points *R* are below this line. This evidences that the gap remains open up to *T*_C_ whereas for higher temperatures the gap is closed. This directly demonstrates the magnetic origin of the gap^[^ ^25^ ^]^ due to the ferromagnetism of the MnSb_2_Te_4_ system.

Comparing the actual experimental data points with the calculated lines reveals that the gap size continuously decreases as the temperature increases and that it closes rather precisely at *T*_C_ = 45–50 K in line with the *T*_C_ obtained by XMCD and SQUID. As described above, the gap size varies spatially across the surface (Figure  3a–c) due to local disorder. Accordingly, at higher temperatures the STS ratios also exhibit a considerable variation depending on where the STS spectra were recorded. For this reason, larger ensembles of data points were recorded at four selected temperatures *T* = 10, 21, 31, and 36 K within an area of 400 nm^2^. From this, the average gap sizes were deduced as $\bar{\Delta }$(21 K) = 11 ± 5 meV, $\bar{\Delta }$(31 K) = 6 ± 1 meV, and $\bar{\Delta }$(36 K) = 4 ± 1 meV, represented by the large gray and white dots in Figure 3d. Thus, the gap indeed gradually shrinks as the temperature approaches *T*_C_ and is closed above, fulfilling precisely the expectations for a ferromagnetic topological insulator.^[^ ^25^ ^]^ Note that the different gray shades of the small data points mark different cooling runs starting from an initial elevated temperature. Hence, during cooling the tip slowly drifts across the sample surface while measuring at varying temperature, thus exploring the variations of Δ versus *T* and spatial position simultaneously. Accordingly, the visible trend of Δ(*T*) relies on the sufficient statistics of probed locations to obtain reliable spatially averaged $\bar{\Delta }$ (large dots).

The conclusion that the gap closes at *T*_C_ is corroborated by comparing the experimental $\bar{\Delta }(T)$ gap evolution with the *M*(*T*) magnetization, based on the relation $\bar{\Delta }(T)\propto M(T)$ derived by theory^[^ ^70^ ^]^ and previous experiments.^[^ ^25^ ^]^ Combining the *M*(*T*) data from SQUID and XMCD (Figure 1d,f and Figure S3, Supporting Information) and the low temperature gap $\bar{\Delta }$(4.3 K) = 17 meV from STS (Figure 3b), $\bar{\Delta }(T)=\bar{\Delta }(4.3\;K)\cdot M(T)/M_{0}$ is obtained and, thus, straightforwardly $\bar{R}(T)$ from the magnetization data. The results are presented as yellow, blue, and red dots in Figure 3d, demonstrating nice agreement with the STS experiments. This further demonstrates the magnetic origin of the gap. Since the center of the gap (Figure S7, Supporting Information) is close to the Dirac point position observed by ARPES (Figure 2e,h), the gap is attributed to the topological surface state, consistent with the out‐of‐plane spin polarization near the Dirac point observed in Figure 2j. Such a magnetic gap of a topological surface state close to *E*_F_ is highly favorable for probing the resulting topological conductivity and its expected quantization.

## 5 DFT Calculations

**Figure 4:**
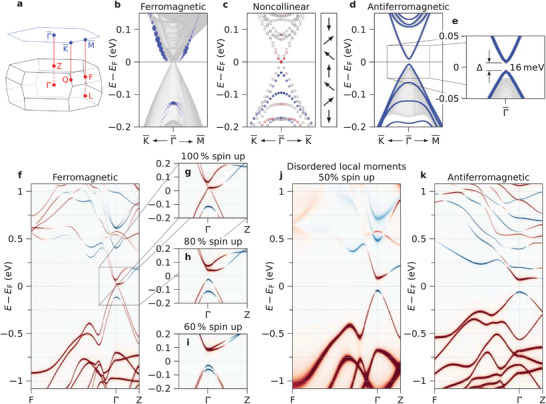
Theoretical predictions for MnSb_2_Te_4_. a) Brillouin zone of the bulk (bottom) and the surface (top) of MnSb_2_Te_4_ with high symmetry points marked. b–e) Band structure from DFT calculations of slab geometries for various magnetic configurations as marked on top. b) Ferromagnetic interlayer coupling calculated for a thick slab. Blue dots: surface states identified via their strength in the top septuple layer. c) Non‐collinear interlayer coupling as sketched to the right by a vector model and calculated for a thin slab. The color code marks the out‐of‐plane spin polarization of the gapped surface‐localized Dirac cone on the background of uncolored bulk‐like states. d) Antiferromagentic interlayer coupling calculated for a slab (blue lines) on top of the projected bulk band structure (gray lines). A gapped Dirac cone appears at $\bar{\Gamma }$ in the slab calculation. e) Zoom into (d) with marked size of the magnetic gap in the Dirac cone (topological surface state). f–k) Band structures from DFT calculation of a bulk geometry for magnetic configurations as marked on top. Color represents the spectral function difference between anion and cation sites for each state with red (blue) representing dominating anion (cation) character (Note S11, Supporting Information). f) Ferromagnetic interlayer coupling exhibiting a topologically protected Weyl cone around Γ. g) Zoom into (f). h,i) same as (g) but with magnetic disorder modelled via overlap of collinear spin‐up and spin‐down states at each Mn site (Note S11, Supporting Information). j) Same as (f), but with full magnetic disorder. (k) Antiferromagnetic interlayer coupling. Note that (h–k) feature an inverted bandgap at Γ as visible by the exchanged colors of the two bands surrounding *E*_F_ rendering these structures topological insulators.

To clarify the origin of the discovered ferromagnetic topological insulator, the electronic band structure of MnSb_2_Te_4_ was calculated by various DFT methods, considering different magnetic configurations, including chemical as well as magnetic disorder (Note S11, Supporting Information). As a general result, the ferromagnetic topological insulator phase is only formed by introducing magnetic disorder. Calculating the bulk band structure, the perfect ferromagnetic system without disorder emerges as a topological Weyl semimetal with a zero bulk bandgap and a Weyl crossing point located along Γ*Z* at about 5% of the Brillouin zone away from Γ (**Figure** **4**f,g, Figures S11 and S12, Supporting Information). While this is in agreement with recent calculations,^[^ ^49^, ^54^ ^]^ it obviously disagrees with our STS and ARPES results. On the other hand, the disorder‐free antiferromagnetic system is found to be a topological insulator with a bulk bandgap of 120 meV (Figure 4k). This is evidenced by the band inversion at Γ in Figure 4k indicated by the color coding of the spectral function difference between cationic and anionic sites [red (blue): dominating anion (cation) character, see Note S11, Supporting Information]. This color code is also used in Figure 4f–k. Slab calculations of the antiferromagnetic surface band structure (Figure 4d) indeed reveal the topological surface state with a gap at the Dirac point due to time reversal symmetry breaking. Discrepancies to earlier calculations^[^ ^52^, ^53^ ^]^ are discussed in Note S11, Supporting Information.

For a more realistic modelling, complex magnetic orders have to be taken into account, deviating from perfect ferromagnetic or antiferromagnetic order as implied by our magnetometry results (Figure 1e, Figure S3, Supporting Information). The extreme case of completely disordered local magnetic moments, without net magnetization, showcases a bulk bandgap of 135 meV with nontrivial topology (Figure 4j) as seen from the band inversion around the gap close to Γ. Varying the degrees of magnetic disorder (Figure 4g–i, Figure S12, Supporting Information) shows that already 20% of magnetic disorder breaks up the Weyl point of ferromagnetic MnSb_2_Te_4_ and opens an inverted bandgap. This topological insulator induced by magnetic disorder is robust against chemical disorder such as the Mn–Sb site exchange that proved to be essential for inducing ferromagnetic order in the system. While a Mn–Sb site exchange of 5% does not affect the band topology, 20% site exchange renders the system trivial (see Figure S11, Supporting Information). The reason is the lowered spin‐orbit interaction in the Sb layers. We have also simulated 7–10% Mn–Sb site exchange plus Mn excess and find that the system is still topological. Our results imply that, contrary to previous conclusions,^[^ ^47^ ^]^ defect engineering can accomplish simultaneously a nontrival band topology and ferromagnetism with high Curie temperature for the MnSb_2_Te_4_ system.

To further assess the robustness of the magnetic gap, slab calculations are employed for various magnetic disorder configurations. The simplest case of a purely ferromagnetic slab does not lead to a Dirac cone, since the bulk bandgap vanishes (Figure 4b). The pure antiferromagnetic order, on the other hand, creates a pronounced Dirac cone with magnetic gap of 16 meV, Figure 4d,e, matching the gap size found by STS (Figure 3b). The magnetic gap size turns out to be a rather local property caused by the exchange interaction in near‐surface MnSb_2_Te_4_ septuple layers. As such, the gapped Dirac cone also forms when the surface of an antiferromagnet is terminated by a few ferromagnetic layers (see Figure S13d, Supporting Information). A relatively strong out‐of‐plane spin polarization at the gap edges (≈60%) is found in that case, matching the results of the spin‐resolved ARPES measured at 30 K (≈25%) nicely, if one takes into account the temperature dependence of the magnetization (Figure 1d,f). For more random combinations of antiferromagnetic and ferromagnetic layers, the Dirac cone with magnetic gap persists, albeit the bulk bandgap vanishes due to the more extended ferromagnetic portions in the structure (see Figure S13a, Supporting Information). Finally, for a system where the magnetic moments of adjacent septuple layers are continuously tilted with respect to each other, a gapped Dirac cone was also observed (see Figure 4c). Likewise, alternate rotations of adjacent collinearly coupled Mn layers by relative angles ≥40° open a gap in the Weyl cone (Figure S12, Supporting Information). Hence, magnetic disorder turns out to be a decisive tool to accomplish topological insulator properties for MnSb_2_Te_4_.

Last but not least, it is noted that the slope of the temperature dependent *M*(*T*) shows a remarkable linear behavior toward *T*_C_, described by an effective critical exponent β = 0.7−1.2. This large β apparently persists for about half of the range between *T* = 0 K and *T*_C_ (Figure 1d,f). Such large β values do not exist in any classical model ranging from β ≃ 0.125 for the 2D Ising model to the mean‐field value of 0.5. The behavior at the classical critical point may, however, strongly change due to quantum fluctuations, which can lead to β = 1 in the presence of disorder^[^ ^71^ ^]^ as experimentally observed.^[^ ^72^, ^73^ ^]^ Note that such disorder is witnessed in our samples by the spatial gap size fluctuations (Figure 3). Moreover, the magnetic phases of MnSb_2_Te_4_ are energetically very close to each other (Table III, Supporting Information)^[^ ^28^ ^]^ which may lead to changes as a function of temperature as suggested by the kink in the *M*(*T*) curve (Figure S3, Supporting Information).

## 6 Summary and Conclusion

Epitaxial MnSb_2_Te_4_ with regularly stacked septuple layers and a slight Mn excess features a robust nontrivial band topology at record‐high Curie temperatures up to *T*_C_ = 50 K. DFT band structure calculations, ARPES and STS experiments showcase a 2D Dirac cone of a topological surface state with a magnetic gap of ≈17 meV close to the Fermi level. The gap disappears at *T*_C_ and above, signifying its magnetic origin. This is corroborated by the out‐of‐plane spin polarization at the Dirac point observed by spin‐resolved ARPES. Ferromagnetism is triggered by the modifications of the exchange interactions induced by Mn–Sb site exchange in combination with a slight in‐plane contraction. Most importantly, excess Mn on Sb sites significantly enforces the ferromagnetic interactions, leading to a factor of two increase of the Curie temperature. Last but not least, the spin‐orbit interaction in MnSb_2_Te_4_ compared to MnBi_2_Te_4_ turns out to be still sufficiently large to maintain both the band inversion and the perpendicular magnetic anisotropy. This renders MnSb_2_Te_4_ highly favorable for the quantum anomalous Hall effect and other topology‐based device applications as the critical temperature is twice as large as for MnBi_2_Te_4_ and the Dirac point is close to *E*_F_ with small spatial variations. Altogether, our results underline that the magnetic properties and interlayer couplings are highly sensitive to the structural disorder in the material and that magnetic disorder is essential to sustain the magnetic topological insulator phase. Indeed, the magnetization features an exotic critical exponent β ≈ 1, which indicates the influence of a quantum critical point, likely merging ferromagnetic and antiferromagnetic order.

## 7 Experimental Section

Details on the sample growth, STEM, RBS, XRD, SQUID magnetometry, resonant scattering and XMCD, ARPES with spin polarimetry, STM and STS, electrical transport measurements, and DFT calculations are provided in the Supporting Information.

## Conflict of Interest

The authors declare no conflict of interest.

## Author Contributions

P.K., J.S.‐B., and S.W. contributed equally to this work, which was coordinated by M.M., G.S., and O.R.

## Supporting information

Supporting Information

## Data Availability

The data that support the findings of this study are available from the corresponding author upon reasonable request.
