# Supplementary material for: Mn‐Rich MnSb2Te4: A Topological Insulator with Magnetic Gap Closing at High Curie Temperatures of 45–50 K
Source: Adv Mater. 2021 Sep 1;33(42):2102935. doi: 10.1002/adma.202102935 (PMC11468489; doi:10.1002/adma.202102935)
Supplement: Supplementary file 1 — Supporting Information [file ADMA-33-2102935-s001.pdf]

© 2021 Wiley-VCH GmbH

ADVANCED MATERIALS

# Supporting Information

for *Adv. Mater.,* DOI: 10.1002/adma.202102935

# Mn-Rich $MnSb_2Te_4$: A Topological Insulator with Magnetic Gap Closing at High Curie Temperatures of 45–50 K

*Stefan Wimmer, Jaime Sánchez-Barriga, Philipp Küppers, Andreas Ney, Enrico Schierle, Friedrich Freyse, Ondrej Caha, Jan Michalička, Marcus Liebmann, Daniel Primetzhofer, Martin Hoffman, Arthur Ernst, Mikhail M. Otrokov, Gustav Bihlmayer, Eugen Weschke, Bella Lake, Evgueni V. Chulkov, Markus Morgenstern, Günther Bauer, Gunther Springholz,* and Oliver Rader**

# Supporting Information

# Mn-rich $MnSb_2Te_4$: A topological insulator with magnetic gap closing at high Curie temperatures of 45–50 K

S. Wimmer,[1] J. Sánchez-Barriga,[2] P. Küppers,[3] A. Ney,[1] E. Schierle,[2] F. Freyse,[2,4] O. Caha,[5] J. Michalička,[6] M. Liebmann,[3] D. Primetzhofer,[7] M. Hoffmann,[8] A. Ernst,[9,10] M. M. Otrokov,[11,12] G. Bihlmayer,[13] E. Weschke,[2] B. Lake,[2] E. V. Chulkov,[14,15,16,17] M. Morgenstern,[3] G. Bauer,[1] G. Springholz,[1] and O. Rader[2]

[1] *Institut für Halbleiter- und Festkörperphysik, Johannes Kepler Universität, Altenberger Straße 69, 4040 Linz, Austria*

[2] *Helmholtz-Zentrum Berlin für Materialien und Energie, Albert-Einstein-Straße 15, 12489 Berlin, Germany**

[3] *II. Institute of Physics B and JARA-FIT, RWTH Aachen University, 52074 Aachen, Germany**

[4] *Institut für Physik und Astronomie, Universität Potsdam, Karl-Liebknecht-Straße 24/25, 14476 Potsdam, Germany*

[5] *Department of Condensed Matter Physics, Masaryk University, Kotlářská 267/2, 61137 Brno, Czech Republic*

[6] *Central European Institute of Technology, Brno University of Technology, Purkyňova 123, 612 00 Brno, Czech Republic*

[7] *Department of Physics and Astronomy, Universitet Uppsala, Lägerhyddsvägen 1, 75120 Uppsala, Sweden*

[8] *Institute for Theoretical Physics, Johannes Kepler Universität, Altenberger Straße 69, 4040 Linz, Austria*

[9] *Institute for Theoretical Physics, Johannes Kepler Universität, Altenberger Straße 69, 4040 Linz, Austria*

[10] *Max Planck Institute of Microstructure Physics, Weinberg 2, 06120 Halle, Germany*

[11] *Centro de Física de Materiales (CFM-MPC), Centro Mixto CSIC-UPV/EHU, 20018 San Sebastián/Donostia, Spain*

[12] *IKERBASQUE, Basque Foundation for Science, 48011 Bilbao, Spain*

[13]*Peter Grünberg Institute and Institute for Advanced Simulation, Forschungszentrum Jülich and JARA, 52425 Jülich, Germany*
[14]*Donostia International Physics Center (DIPC), 20018 San Sebastián/Donostia, Spain*
[15]*Departamento de Física de Materiales, Facultad de Ciencias Químicas, Universidad del País Vasco, Apdo. 1072, 20080 San Sebastián/Donostia, Spain*
[16]*Saint Petersburg State University, 198504, Saint Petersburg, Russia*
[17]*Tomsk State University, Tomsk, 634050, Russia*

(Dated: June 29, 2021)

## CONTENTS


S1. Sample Growth 4

S2. Electron Microscopy 5

S3. Rutherford Backscattering Spectrometry 5

S4. X-ray Diffraction 5

S5. Magnetometry by SQUID 8

S6. Comparison with Magnetic Properties of Other $MnSb_2Te_4$ Samples 9

S7. Resonant Scattering and X-ray Circular Dichroism 10

S8. ARPES and spin-resolved ARPES 11

S9. Scanning Tunneling Microscopy and Spectroscopy 15
- A. Measurement Details 15
- B. Band Gap Determination at 4.3 K 16
- C. $dI/dV(V)$ Curves at Different Temperatures 17
- D. Determination of Band Gaps at Elevated Temperature 19

S10. Electric Transport Measurements 22

S11. Density Functional Theory Calculations 22
- A. Details of the Calculations 22
- B. Magnetic Ground State for Different Disorder Configurations 25
- C. Topological Properties for Different Disorder Configurations 26
- D. Influence of Charge Doping on the Magnetic Interactions 29

References 32



* These authors contributed equally to this work

The following Supporting Notes are ordered according to the methods applied. Each Supporting Note describes both the details of the experiment or the calculations and additional data supporting the conclusions from the main text.

## S1: SAMPLE GROWTH

$MnSb_2Te_4$ films were grown by molecular beam epitaxy (MBE) on $BaF_2(111)$ substrates using a Varian GEN II system. Compound $Sb_2Te_3$ and elemental Mn and Te sources were employed for control of stoichiometry and composition. The Mn:$Sb_2Te_3$ flux ratio was set to 1:1 in order to obtain the nominal 1:2:4 stoichiometry of the $MnSb_2Te_4$ phase. The corresponding Mn and $Sb_2Te_3$ flux rates were 0.1 monolayers (ML)/s and 0.1 quintuple layers/s, respectively, determined by the quartz microbalance method with precision of $\pm 5\%$. In addition, an excess Te flux of 1.5 ML/s was used. Typical sample thicknesses were 200 nm. Deposition was carried out at a sample temperature of 290°C at which perfect 2D growth is sustained independently of the Mn concentration, as verified by *in situ* reflection high energy electron diffraction and atomic force microscopy. At the given substrate temperature of 290°C, the incorporation coefficient is unity for Mn due to its very low vapour pressure, but for $Sb_2Te_3$ a small fraction desorbs during growth and is thus, not incorporated. This leads to a slightly Mn rich composition of the epilayers as revealed by Rutherford backscattering spectrometry (RBS) described in Supporting Note S3. The composition was further checked by x-ray diffraction analysis described in Supporting Note S4. Only samples exhibiting the diffraction spectra of the $MnSb_2Te_4$ phase were used for further investigations.

For angle-resolved photoelectron spectroscopy (ARPES) performed at BESSY II and scanning tunneling microscopy (STM) performed at RWTH Aachen University, samples were transferred from the MBE system using an ultrahigh vacuum (UHV) suitcase operated in the low $10^{-10}$ mbar pressure range that allowed transportation without breaking UHV conditions. Samples used for magnetometry and x-ray magnetic circular dichroism (XMCD) were capped *in situ* after MBE growth with amorphous Se and Te capping layers to protect the surface against oxidation.

## S2: ELECTRON MICROSCOPY

High-resolution scanning transmission electron microscopy (TEM) was performed with a FEI Titan 60-300 Themis instrument equipped with a Cs image corrector. The TEM data were recorded with a high-angle annular dark field (HAADF) detector and the images processed using a fast Fourier transform and Fourier mask filtering technique for noise minimization. Thin cross-sectional lamellae from $MnSb_2Te_4$ films were prepared by focused ion beam milling (FEI Helios NanoLab 660).

## S3: RUTHERFORD BACKSCATTERING SPECTROMETRY

The chemical composition of the samples was determined by Rutherford backscattering spectrometry (RBS) employing a primary beam of 2 MeV $^4He^+$ ions provided by the 5 MV 15SDH-2 Pelletron accelerator at Uppsala University. A solid-state detector was placed in backscattering geometry under an angle of 170° with respect to the primary beam. The beam incidence angle was randomized in a 3° angular interval around an equilibrium angle of 18° with respect to the surface normal to counteract potential channeling effects. The resulting spectra (Fig. S1) were fitted by the SIMNRA software package [1] to obtain the film composition and confirm its uniformity over the film cross section. Analysis of the RBS data as shown in Fig. S1 yields an excellent fit (blue line in Fig. S1) for atom concentrations of 57.0% Te, 27.7% Sb and 15.3% Mn with a high uniformity throughout the film cross section. The obtained composition of $Mn_{1.06}Sb_{1.94}Te_4$ is close to the nominal $MnSb_2Te_4$ stoichiometry, but features a slight Mn excess of $\sim 6\%$ and a slight Sb deficiency of $\sim 3\%$ , indicating a small amount of substitution of Sb atoms by additional Mn atoms in addition to Mn-Sb site exchange, as corroborated by x-ray diffraction analysis shown in Supporting Note S4.

## S4: X-RAY DIFFRACTION

The crystalline structure was determined by x-ray diffraction (XRD) scans and reciprocal space maps recorded in the vicinity of the $(10\overline{1}.20)$ reciprocal lattice point. The measurements were performed using a Rigaku SmartLab diffractometer with Cu x-ray tube and channel-cut Ge(220) monochromator. The results are shown as red lines in Fig. S2. The

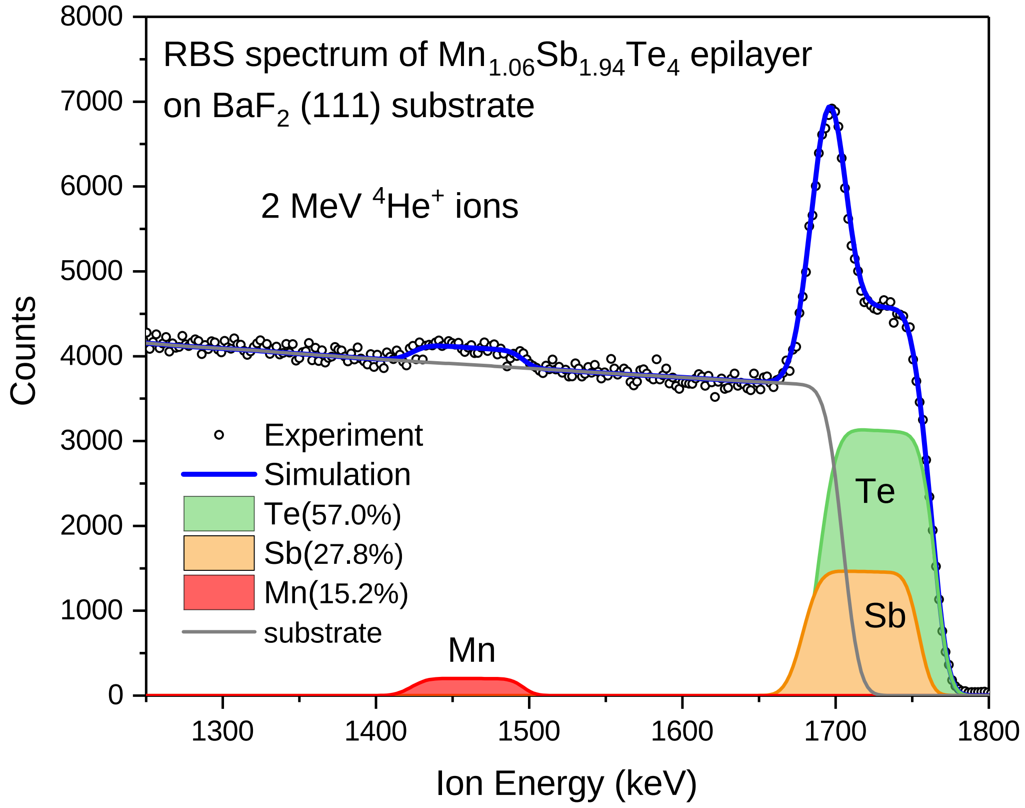


Figure S1. **Chemical composition and depth profile.** Rutherford backscattering spectrum measured in random geometry. The simulation of the total backscattering signal using SIMNRA (blue line) is in excellent agreement with the experimental data (open circles). The corresponding contributions of the film constituents (Te, Sb and Mn) are indicated by the green, orange and red lines and correspondingly shaded areas. The data reveal a slight Mn excess of $\sim 6\,\%$ and an Sb deficiency of $\sim 3\,\%$ with respect to the ideal $MnSb_2Te_4$ stoichiometry, suggesting a partial Mn replacement of Sb atoms in the Sb layer. This yields a composition of $Mn_{1.06}Sb_{1.94}Te_4$ of the films.

symmetric scans along the [000.1] reciprocal space direction ($c$-axis) were fitted with a modified one dimensional paracrystal model [2, 3] (black lines in Fig. S2). We have fitted the data of two samples and obtained consistent results of $a = 4.23$ Å and $c = 40.98$ Å.

The Mn-Sb site exchange was modelled by assuming various fractional occupancies of Mn on Mn sites $C_{Mn/Mn}$ in the central cation lattice plane of the septuples, ideally occupied by Mn only, the rest of these sites being occupied by Sb atoms. This accordingly leads to

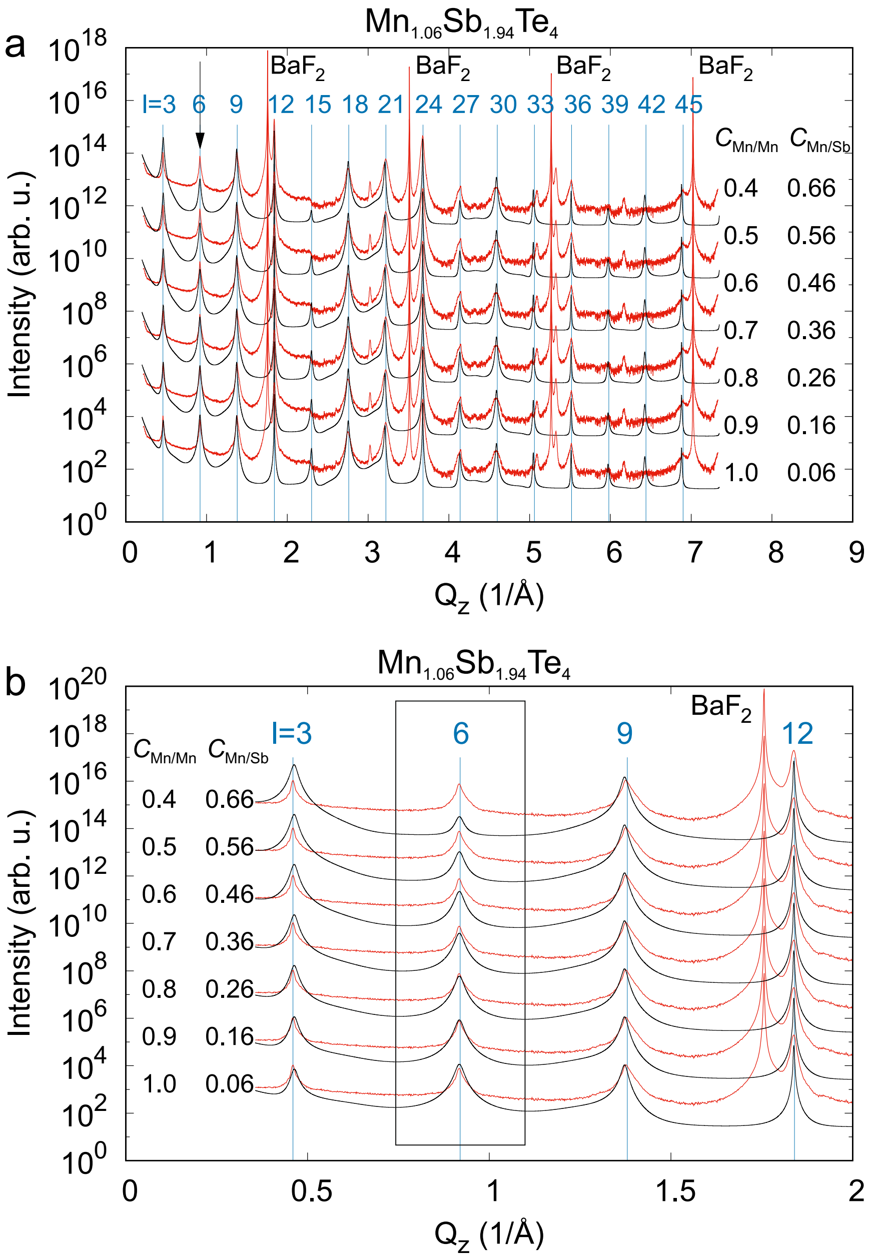


Figure S2. **X-ray diffraction spectra with fits.** (a) Experimental x-ray diffraction spectrum of the $MnSb_2Te_4$ epilayer (red lines) compared with simulations (black lines) using a one-dimensional paracrystal model [2, 3] while assuming different Mn occupation in the central layer ($C_{Mn/Mn}$) and in the adjacent nominal Sb layers ($C_{Mn/Sb}$) of the septuple. Blue lines denote positions of (0 0 0 $l$) reciprocal lattice points. (b) Zoom into (a) showcasing the strong sensitivity of the peak at $Q_z \approx 0.9$ Å$^{-1}$ (black arrow in (a)) to the Mn-Sb site exchange. The concentration pairs $C_{Mn/Mn}$ and $C_{Mn/Sb}$ are chosen to correspond to the $Mn_{1.06}Sb_{1.94}Te_4$ stoichiometry determined by RBS. Peaks of the $BaF_2$ substrate are indicated.

a partial occupancy of the Sb sites in the outer cation lattice planes by Mn atoms with $C_{Mn/Sb} = (C_{tot} - C_{Mn/Mn})/2$ where $C_{tot}$ is the total number of Mn atoms in each septuple. The factor 1/2 arises from the fact that the exchanged Mn from the center of the septuple is evenly distributed between the two outer Sb layers. In the case of excess Mn in the layers as seen by RBS, the sum $(C_{Mn/Mn} + C_{Mn/Sb}) > 1$.

With these definitions, the structure factor of $MnSb_2Te_4$ reads

$$F(Q_z) = \sum_n C_n f_n(Q_z) \exp(-iQ_z z_n), \tag{1}$$

where $f_n(Q_z)$ is the atomic form factor of the $n$-th atom in the unit cell, $z_n$ its coordinate, $C_n$ its occupancy, $Q_z$ its position in reciprocal space and the summation runs over all atoms in the septuple layer. For Sb, Te, and Mn the atomic form factors at $Q_z = 0.9\,\text{Å}^{-1}$ are 48.9, 49.9 and 23.6, respectively.

The comparison of the measured diffraction spectrum with the simulation for different amounts of Mn-Sb site exchange are shown in Fig. S2, taking into account the small Mn excess in the layers ($C_{Mn/Mn} + C_{Mn/Sb} = 1.06$ ) as derived by RBS (Fig. S1). $C_{Mn/Mn} = 0.4$ and $C_{Mn/Sb} = 0.66$ at the top of the figure corresponds to 40% Mn atoms in the center and 66% in the outer two cation layers of the septuple. Figure S2(b) highlights the low $Q_z$ region, evidencing the strong sensitivity of the [000.6] peak at $Q_z = 0.9\,\text{Å}^{-1}$ to the Mn and Sb exchange. For this peak, the two different cation layers contribute to the structure factor with almost exactly opposite phase, while the contribution of the Te layers almost cancels. Because of the different Mn and Sb atomic form factors, the intensity of this peak rapidly diminishes with increasing Mn-Sb exchange and completely vanishes for complete intermixing, i.e., $C_{Mn/Mn} \simeq C_{Mn/Sb}$. The best fit to the experiment is found for $C_{Mn/Mn}$ between 0.9 and 0.8 and $C_{Mn/Sb} \sim 0.2$, evidencing a non-ideal distribution of Mn and Sb in the respective layers.

## S5: MAGNETOMETRY BY SQUID

The magnetic properties were determined by a superconducting quantum interference device (SQUID) magnetometer (Quantum Design MPMS-XL) as a function of temperature from 2 K to 300 K. The external field **H** was applied either parallel (in plane) or perpendicular (out of plane) to the epilayer surface, that itself is oriented perpendicular to the

crystallographic $c$ axis. The diamagnetic contribution of the substrate was determined from the slope of the magnetization $M(H)$ recorded at high magnetic fields and 300 K well above the Curie temperature $T_C$ of $MnSb_2Te_4$, where the magnetic contribution of the thin film is completely superseded by that of the thousand times thicker substrate. The derived substrate contribution was then subtracted from the raw data recorded at lower $T$. Identical sample pieces were used for in-plane and out-of-plane measurements.

Figure S3 shows out-of-plane $M(T)$ data while field cooling at 10 mT for three different samples. They reveal a quite similar $T_C$ and consistently display a ferromagnetic behavior via its remanence. Data for sample 1 are also shown in Fig. 1 (main text) where they give a slightly smaller $T_C$ within the error bars.

Figure S3 (top) features a small kink at 20–25 K that indicates deviations from pure ferromagnetic order possibly indicating a second phase transition that might contribute to the large critical exponent $\beta \simeq 1$ as discussed in the main text and attributed there to the vicinity to a quantum critical point.

## S6: COMPARISON WITH MAGNETIC PROPERTIES OF OTHER $MNSB_2TE_4$ SAMPLES

Table I compares the results of our three $MnSb_2Te_4$ samples with data from the literature. The comparison highlights the exceptionally large Curie temperature of the Mn-rich epitaxial films.

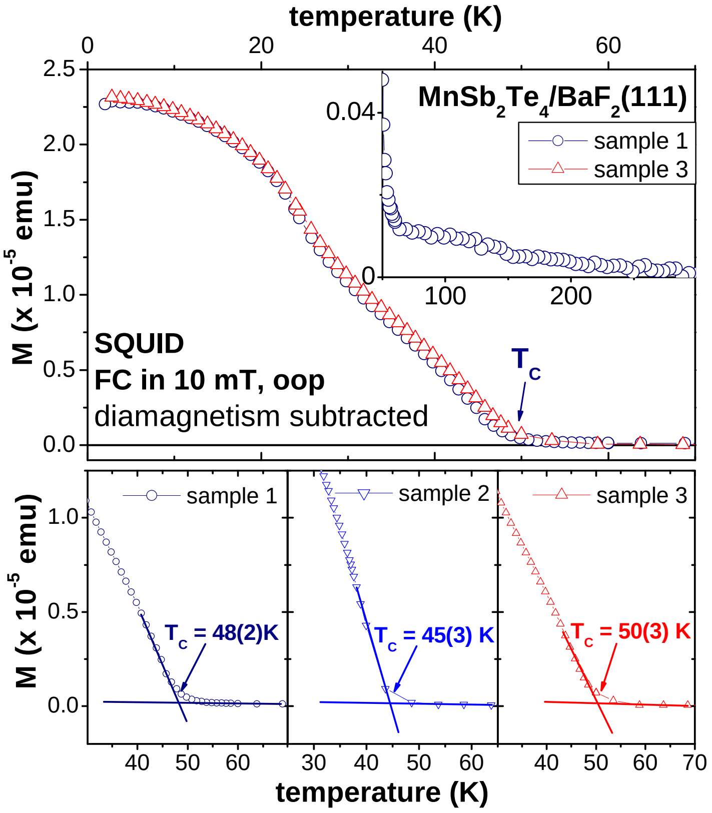


Figure S3. **Magnetometry by SQUID.** Magnetization $M(T)$ of three individual $MnSb_2Te_4$ epilayers measured by SQUID. $M(T)$ was measured in out-of-plane (oop) direction while field cooling (FC) in a field of 10 mT. Linear extrapolation to $M = 0$ emu was used to estimate $T_C$ as marked. Notice the small kink in $M(T)$ around 20 K in the top panel which indicates deviations from pure ferromagnetic order. Inset: Full temperature dependence of the magnetization $M(T)$ up to 300 K on a vertical scale enlarged by a factor of 40. From the SQUID raw data, the diamagnetic contribution from the $BaF_2$ substrate of 0.0205 $\mu$emu/Oe was substracted.

## S7: RESONANT SCATTERING AND X-RAY CIRCULAR DICHROISM

Resonant scattering and XMCD were measured at the extreme ultraviolet (XUV) diffractometer of the UE46-PGM1 undulator beam line of BESSY II at Helmholtz-Zentrum Berlin. The XMCD signal was obtained by measuring the difference of the (0001) Bragg peak intensities for incident photons with opposite circular polarization and the photon energy tuned to the Mn-$L_3$ resonance. In this setup, the sample was field cooled down to 10 K in an external field of about 0.5 T provided by a removable permanent magnet. Subsequent po-

| nominal stoichiometry | growth | magnetism | critical temperature | reference |
|---|---|---|---|---|
| $MnSb_2Te_4$ | epitaxial film | FM | $T_C$=46-48 K | sample 1, this work |
| $MnSb_2Te_4$ | epitaxial film | FM | $T_C$=45 K | sample 2, this work |
| $MnSb_2Te_4$ | epitaxial film | FM | $T_C$=50 K | sample 3, this work |
| $MnSb_2Te_4$ | single crystal | AFM | $T_N$= 19 K | J.-Q. Yan et al. [a]) |
| $MnSb_2Te_4$ | single crystal | AFM | $T_N$=19.5 K | Y. Chen et al. [b]) |
| $MnSb_{1.8}Bi_{0.2}Te_4$ | single crystal | FM | $T_C$=26 K | Y. Chen et al. [b]) |
| $MnSb_2Te_4$ | polycrystal | FM | $T_C$=25 K | Murakami et al. [c]) |

Table I. **Magnetic properties of $MnSb_2Te_4$** for epitaxial films, bulk crystals and polycrystals, comparing the ferromagnetic (FM) Curie temperatures $T_C$ determined in the present work to $T_C$ and antiferromagnetic (AFM) Néel temperatures $T_N$ from the literature. [a]) J.-Q. Yan et al., Phys. Rev. B 100, 104409 (2019); [b]) Y. Chen et al., Phys. Rev. Mat. 4, 064411 (2020); [c]) T. Murakami et al., Phys. Rev. B 100, 195103 (2019).

larization dependent and wave-vector dependent measurements were performed in zero field at various temperatures. The recorded XMCD data are thus a direct measurement of the remanent ferromagnetic polarization of the Mn moments in $MnSb_2Te_4$.

## S8: ARPES AND SPIN-RESOLVED ARPES

ARPES measurements were performed at 30 K with a Scienta R4000 hemispherical analyzer at the RGBL-2 end station of the U125/2 undulator beamline at BESSY II. Light is incident under an azimuthal angle of 45° and a polar angle of 90° with respect to the sample surface. The light polarization is linear and horizontal. Photon energies between 19 and 70 eV were employed. Spin-resolved ARPES spectra were acquired with a Mott-type spin polarimeter operated at 25 kV, capable of detecting both in-plane and out-of-plane spin components. Samples were transported to the RGBL-2 setup in a UHV suitcase to always maintain pressures below $1 \cdot 10^{-10}$ mbar. Overall resolution of ARPES measurements was 10 meV (energy) and 0.3° (angular). Resolutions for spin-resolved ARPES were 45 meV

(energy) and 0.75 ° (angular).

For the measurement in Fig. 2j, main text, the sample was magnetized in situ at 30 K by applying a pulsed magnetic field from a removable coil of $\sim \pm 0.5$ T in the direction perpendicular to the surface. As usual, the spin-resolved ARPES experiment is conducted in remanence. The movement between the ARPES chamber and the preparation chamber, where the coil is situated, is a vertical movement of the cryostat so that the sample is always at 30 K.

Figure S4 shows a complete set of spin-polarized ARPES data featuring all three orthogonal spin directions for a finite $k_{\parallel} = \pm 0.15\,\text{Å}^{-1}$. Only in-plane spin polarization perpendicular to the electron wave vector $k_{\parallel}$ appears and reverses sign with a sign change of $k_{\parallel}$. This evidences a spin texture that rotates anti-clockwise (right-handed) around the measured Dirac cone (Fig. 2g,i, main text) with the electron spin locked to the electron momentum. Such helical in-plane spin texture is a key signature of the topological character of the Dirac cone. Note that the ferromagnetism of $MnSb_2Te_4$ exhibits out-of-plane anisotropy such that bulk states cannot have in-plane spin polarization, which fully supports the assignment to the Dirac cone surface state. To deduce the Dirac point energy $E_D$, the peaks within momentum distribution curves are fitted by two Lorentzians (Fig. S4e) and the energy dependent peak maxima are connected via two fitted lines (Fig. S4d and Fig. 2d,h, main text) that cross at $E_D - E_F = 20 \pm 7\,\text{meV}$. The binding energy range of the fit was determined by the possibility to fit separate Lorentzians, see Fig. S4d,e. Since we do not observe any surface band bending by residual gas adsorption (prominent for the n-doped systems such as $Bi_2Se_3$ and $Bi_2Te_3$), the Dirac point can be regarded as a rather good measure of the chemical potential.

Figure S5 shows that the Dirac cone surface state has no dispersion with the wave vector perpendicular to the surface plane, in contrast to the bulk valence band. Figure S5 is the input for Fig. 2b, main text, with linear interpolation between the 9 photon energies. Table II shows the momentum values in kinetic energy.

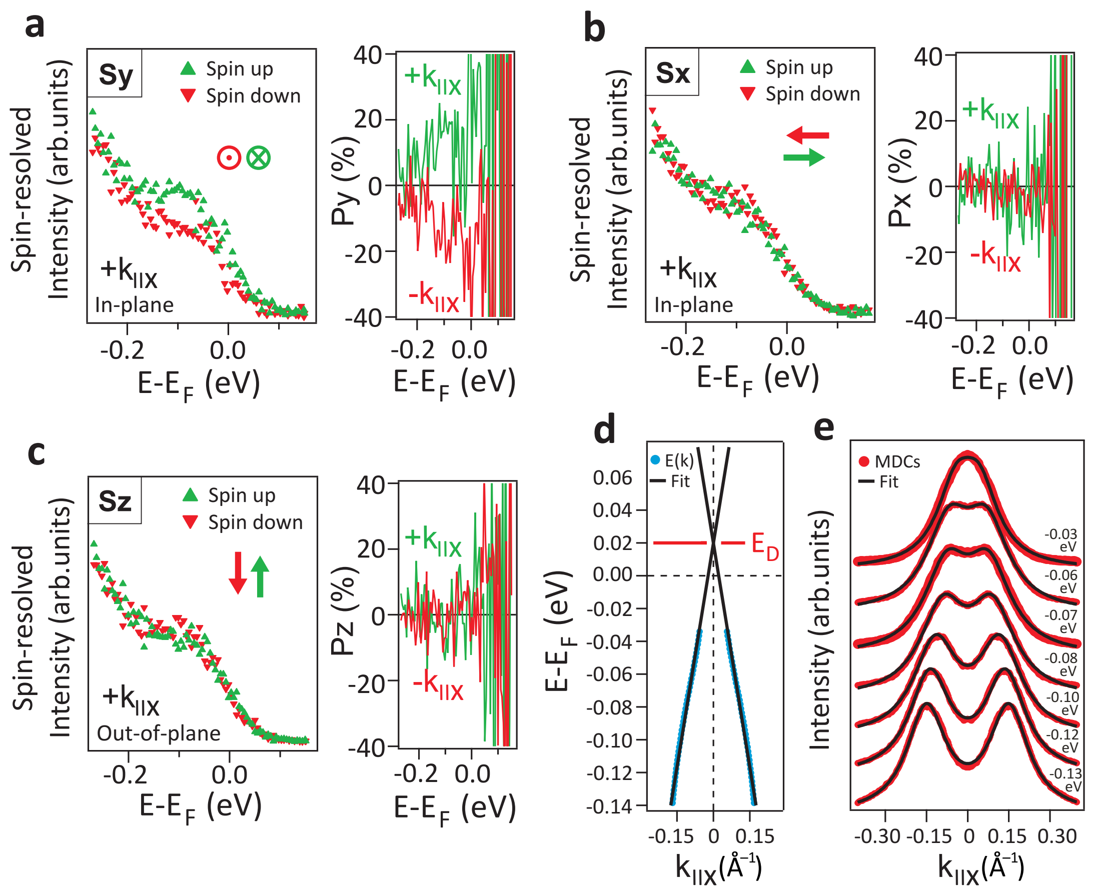


Figure S4. **Spin-resolved ARPES data and fit of Dirac cone.** All three spin polarization components are shown for $k_{\parallel,x} \simeq \pm 0.15\,\text{Å}^{-1}$ (indicated in Fig. 2h, main text). Left hand side of each subfigure shows the ARPES data for the two opposite spin channels at $k_{\parallel,x} \simeq +0.15\,\text{Å}^{-1}$, while the right hand side shows the resulting spin polarization $P_i$ for both $k_{\parallel,x} \simeq \pm 0.15\,\text{Å}^{-1}$. (a) In-plane spin components perpendicular to $k_{\parallel,x}$ (same as Fig. 2g, main text). (b) In-plane spin components parallel/antiparallel to $k_{\parallel,x}$. (c) Out-of-plane spin components. The symbols for spin direction and orientation are relative to panel (d). (d) The Dirac energy $E_\mathrm{D}$ is determined by the crossing of two lines given by fits to experimental data points which, in turn, were determined by Lorentzians fitted to momentum distribution curves (MDC). These fits are shown in panel (e). $h\nu = 25$ eV.

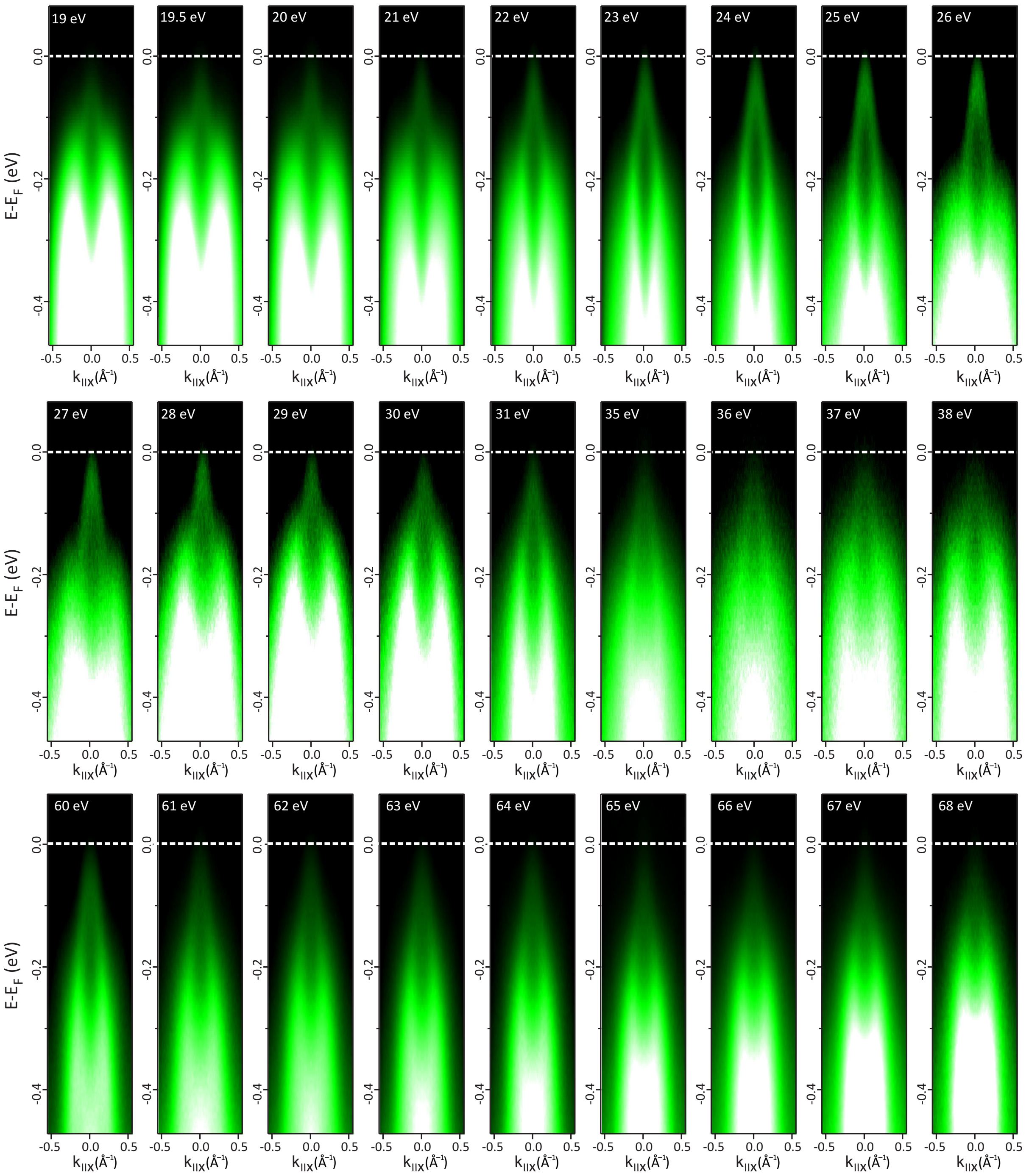


Figure S5. **Photon-energy-dependent ARPES data.** Energy-momentum dispersions for photon energies from 19 to 68 eV showing a 2D Dirac cone and the dispersion of 3D bulk states with photon energy. The periodicity in the bulk dispersion demonstrates that the bulk Brillouin zone is probed completely.

| hν (eV) | $k_z$(Å$^{-1}$) |
|---|---|
| 60 | 4.341 |
| 61 | 4.371 |
| 62 | 4.401 |
| 63 | 4.431 |
| 64 | 4.460 |
| 65 | 4.490 |
| 66 | 4.519 |
| 67 | 4.548 |
| 68 | 4.576 |

Table II. **Perpendicular momentum.** Determination of the perpendicular momentum for the data shown in Fig. 2a,b, main text. Distance from Γ to Z corresponds to 0.1327 Å$^{-1}$. An inner potential of 11.8 eV was used.

## S9: SCANNING TUNNELING MICROSCOPY AND SPECTROSCOPY

### A: Measurement Details

STM measurements were conducted in a home built UHV-STM operating down to $T = 4.3\,\text{K}$. Cr tips were firstly etched ex-situ and additionally prepared in UHV by field emission on clean W(110). Topography images were recorded in constant-current mode at a tunneling current $I$ and bias voltage $V$ applied to the sample. The $\mathrm{d}I/\mathrm{d}V(V)$ spectra for scanning tunneling spectroscopy (STS) were recorded after firstly stabilizing the tip-sample distance at voltage $V_{\text{Stab}} = -0.1\,\text{V}$ and current $I_{\text{Stab}} = 0.1\,\text{nA}$, if not mentioned differently in the captions. Afterwards, the feedback loop was opened and $\mathrm{d}I/\mathrm{d}V$ was recorded using standard lock-in technique with modulation frequency $f = 1219\,\text{Hz}$ and amplitude $V_{\text{mod}} = 1.4\,\text{mV}$ while ramping $V$. The spectra were normalized to account for remaining vibrational noise during stabilization by equilibrating the integral between $V_{\text{Stab}}$ and $V = 0\,\text{mV}$. We crosschecked that $dI/dV$ curves barely depend on the chosen $I_{\text{stab}}$ and, hence, on the tip-surface distance (Fig. S6).

For measurements above the base temperature of 4.3 K, the STM body was exposed

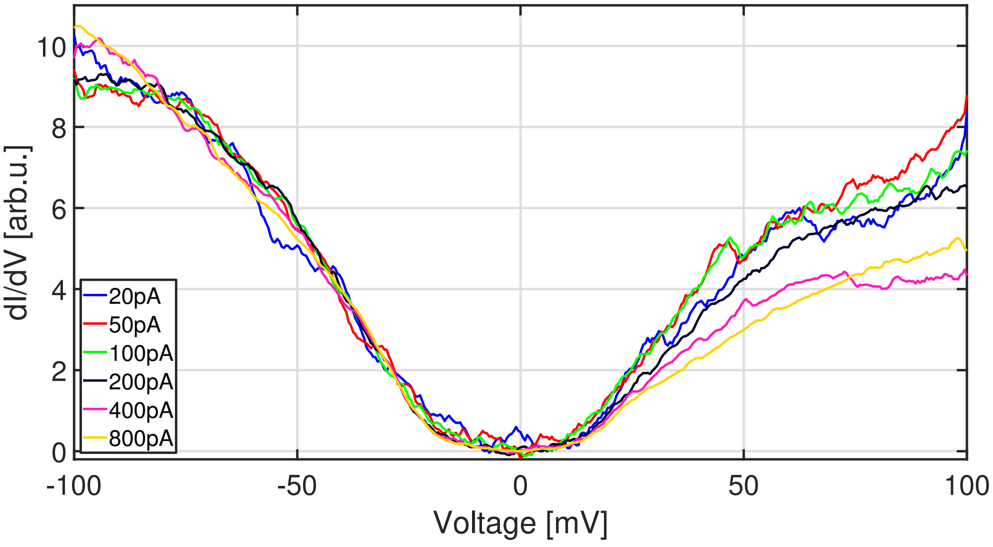


Figure S6. **STS curves recorded at different set points.** $dI/dV(V)$ curves recorded at the same position after stabilizing the tip at $V_{\text{stab}} = -100\,\text{mV}$ and various $I_{\text{stab}}$ as marked, $T = 4.3\,\text{K}$.

to thermal radiation via opening of a radiation shield until a maximum $T \simeq 60\,\text{K}$ was achieved. Then, the shield was closed, and $\text{d}I/\text{d}V(V)$ curves were recorded while the sample temperature slowly decreased back to 4.3 K. Measurements at constant $T > 4.3\,\text{K}$ were performed with partly open shield. A more frequent stabilization between subsequent $dI/dV$ curves was necessary due to the remaining thermal drift of the tip-sample distance during the cooling process. This implies shorter recording times that have been compensated by a more intense averaging of subsequently recorded curves.

### B: Band Gap Determination at 4.3 K

The band gaps at $T = 4.3\,\text{K}$ were determined as follows. First, the noise level of the $dI/dV$ curves was reduced by averaging $3 \times 3$ curves covering an area of $(1.2\,\text{nm})^2$. Subsequently, an averaging of $dI/dV(V)$ across $\pm 2\,\text{mV}$ in bias direction was employed. Then, we estimated the remaining $dI/dV$ noise level as the maximum of $|dI/dV|$ that appears with similar strength positively and negatively. For that purpose, we employed about 50 randomly selected $dI/dV(V)$ curves. Afterwards, the threshold was chosen slightly above the determined noise level. This threshold is marked in Fig. 3a #1 as dashed line and in Fig. 3c as yellow line in the color code bar. The voltage width, where the $dI/dV(V)$ spectra stayed below this threshold, defines the measured gap $\Delta$ as used in Fig. 3a–c, main text.

We crosschecked that $dI/dV$ values below the noise level appeared exclusively close to the determined band gap areas. The resulting gap size $\Delta$ turned out to barely depend on details of the chosen noise threshold.

Figure S7a and b display two additional $\Delta(x, y)$ maps like the ones in Fig. 3b, main text, but recorded on different areas of the sample surface. They exhibit a similar range of spatial fluctuations of $\Delta$ as shown in Fig. 3b, main text. Sometimes, $\Delta$ could not be determined from the $dI/dV(V)$ curves as, e.g., in the bright area marked by a black circle in Fig. S7a. There, the spectra stayed below the threshold on the positive $V > 0\,\mathrm{mV}$ side up to $100\,\mathrm{mV}$ while a gap edge is observed only on the negative side, for an unknown reason. These spectra ($\sim 5\,\%$ of all spectra) are discarded from further analysis including the histogram of Fig. 3b, main text. The correlation length $\xi$ of $\Delta(x, y)$ is calculated as FWHM of the correlation function resulting in $\xi \simeq 2\,\mathrm{nm}$ as given in the main text.

The central energy within the gap, $E_0$, is deduced as the arithmetic mean of all voltages where the $\mathrm{d}I/\mathrm{d}V$ signal remains below the threshold. Figure S7c–f displays maps $E_0(x, y)$ for the three studied areas as well as a resulting $E_0$ histogram. Favorably, the average of $E_0$ is rather precisely at $E_\mathrm{F}$ showing only small spatial fluctuations in the meV range. The small discrepancy of the average $E_0 \simeq E_\mathrm{F}$ found by STS to the Dirac point determined by ARPES ($20\,\mathrm{meV}$ above $E_\mathrm{F}$) might be due to the different recording temperatures ($4.3\,\mathrm{K}$ vs. $300\,\mathrm{K}$) or to sample to sample variations.

#### C: $dI/dV(V)$ Curves at Different Temperatures

Figure S8 displays $dI/dV(V)$ spectra recorded at various temperatures as used for the band gap evaluation displayed in Fig. 3d, main text. An obvious band gap with a voltage region of $dI/dV \simeq 0\,\mathrm{nS}$ is only found up to about $30\,\mathrm{K}$. At larger $T$, the curves partly stay close to $dI/dV = 0\,\mathrm{nS}$ around $E_\mathrm{F}$, but partly strongly deviate from $dI/dV = 0\,\mathrm{nS}$. This varying behaviour requires a more detailed analysis to determine $\Delta$, since a small deviation from $dI/dV = 0\,\mathrm{nS}$ might also be caused by Fermi level broadening of the tip that probes a local density of states (LDOS) of the sample with a small band gap.

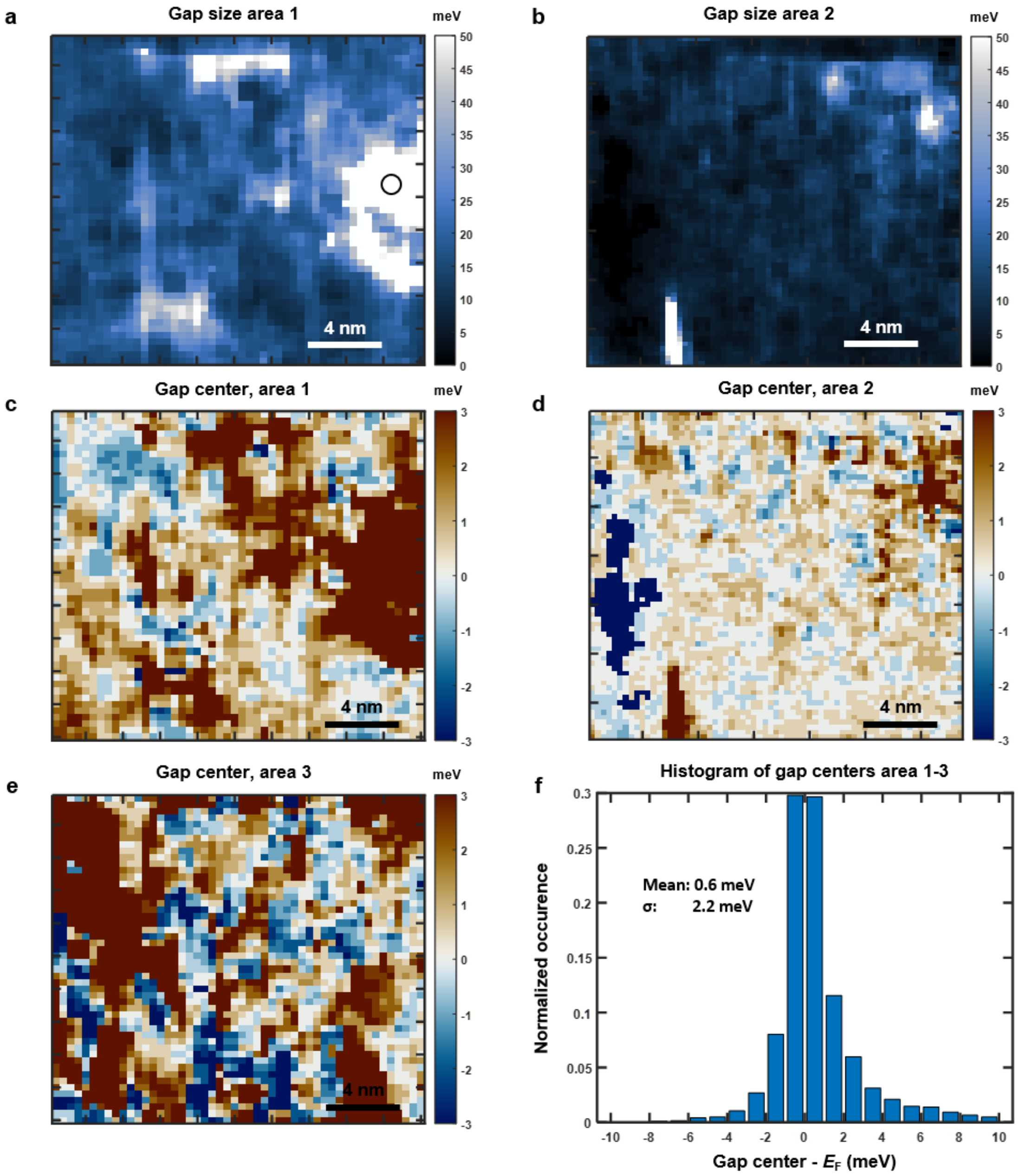


Figure S7. **Maps of gap size $\Delta$ and gap center $E_0$ at 4.3 K.** (a,b) Spatial maps of the gap size $\Delta(x, y)$ as obtained from $dI/dV(V)$ curves recorded at $T = 4.3$ K. Two additional surface areas named area 1 and area 2 are shown, while area 3 is displayed in Fig. 3b, main text. The black circle marks a region, where the determination of a band gap was not possible (see text). (c)-(e) Spatial maps of the center of the gap $E_0(x, y)$ with respect to the Fermi level $E_F$ for the 3 different areas. (f) Histogram of $E_0$ for all three maps with marked mean and standard deviation $\sigma$.

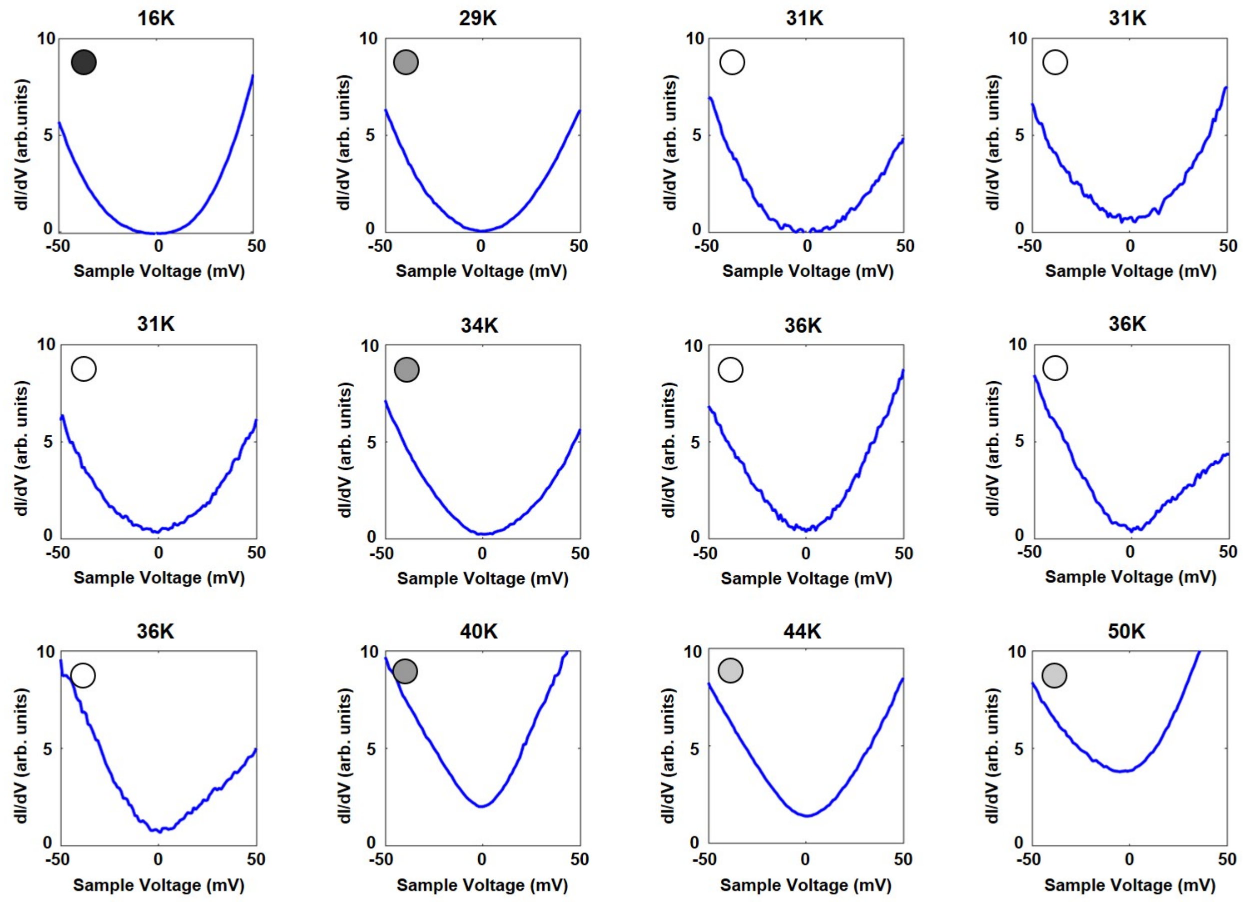


Figure S8. **STS spectra recorded at different temperatures.** Selected d$I$/d$V$(V) curves ordered with increasing $T$ as marked on top. The dot in the upper left is colored identically to the extracted band gap from the same curve as displayed in Fig. 3d, main text.

### D: Determination of Band Gaps at Elevated Temperature

The requirement of a more detailed analysis is demonstrated in Fig. S9a. A $dI/dV(V)$ spectrum exhibiting a gap size $\Delta = 17\,\mathrm{meV}$ is displayed (blue line) as recorded at 4.3 K. This spectrum is then convolved with the derivative of the Fermi-Dirac distribution function, proportional to $1/\cosh^2(eV/2k_\mathrm{B}T)$ [4] (other colored lines in Fig. S9a). By this convolution, we mimick the expected appearance of the same spectrum at elevated $T$. Obviously, the previously introduced method of gap determination would obtain $\Delta = 0\,\mathrm{meV}$ for all $T \geq 31\,\mathrm{K}$, albeit the LDOS of the sample still exhibits $\Delta = 17\,\mathrm{meV}$.

In order to deduce the correct $\Delta$ from $dI/dV(V)$ curves recorded at such large $T$, we introduce the ratio $R = [\mathrm{d}I/\mathrm{d}V(0\,\mathrm{mV})]/[\mathrm{d}I/\mathrm{d}V(-50\,\mathrm{mV})]$ that turns out to be monotonously

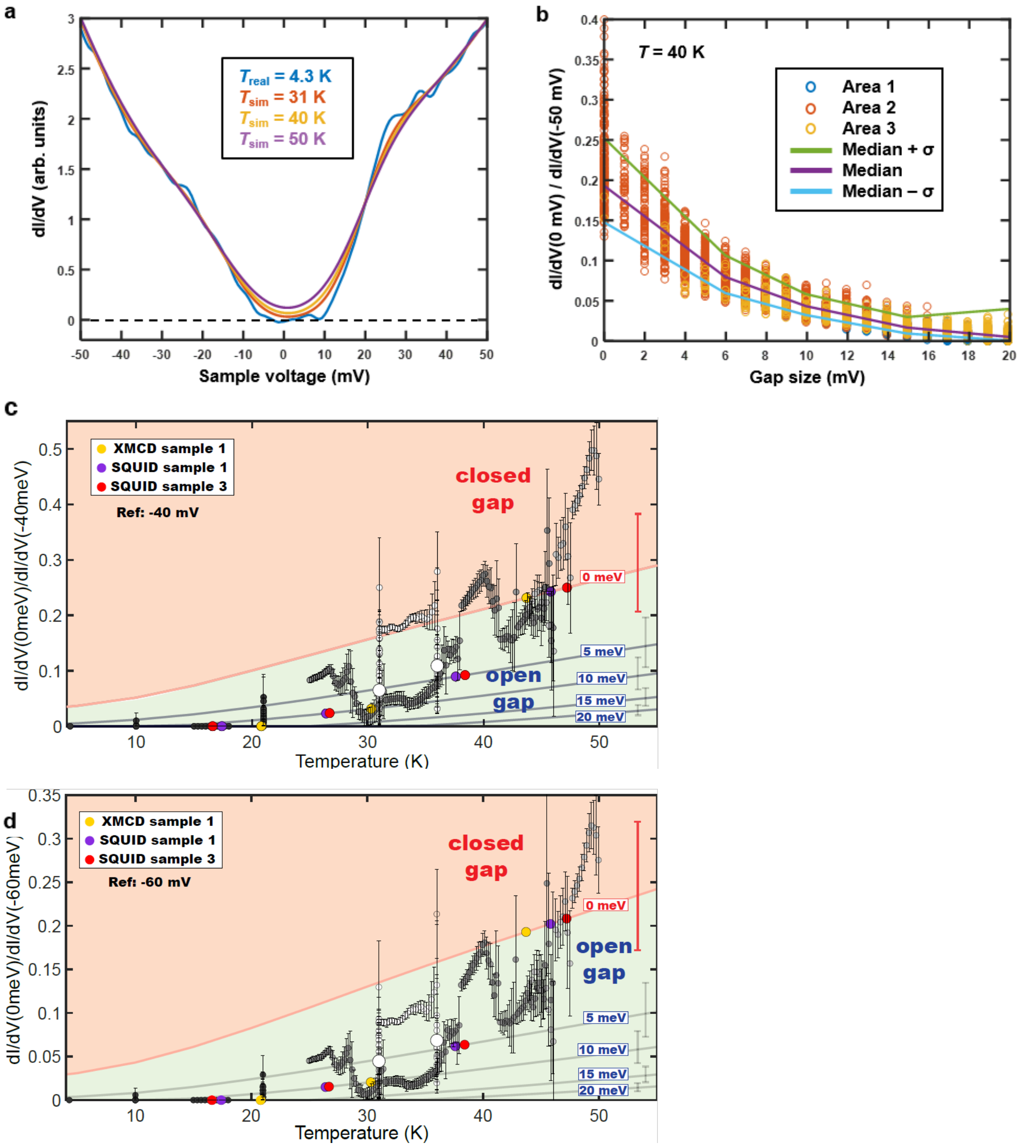


Figure S9. **Gap size determination at elevated $T$.** (a) $dI/dV(V)$ spectrum with gap $\Delta = 17\,\text{meV}$ recorded at 4.3 K (blue) and the same spectrum after convolution with the derivative of the Fermi-Dirac distribution function for $T = 31\,\text{K}$ (red), $T = 40\,\text{K}$ (yellow) and $T = 50\,\text{K}$ (violet). The Fermi level broadening leads to an apparently ungapped $dI/dV$ curve, albeit the sample LDOS features a gap of 17 meV. (b) Relation between the measured gap size $\Delta$ of $dI/dV(V)$ curves recorded at 4.3 K and the ratio $R = [dI/dV(V = 0\,\text{mV})]/[dI/dV(V = -50\,\text{mV})]$ of the same $dI/dV(V)$ curves deduced after convolution with the derivative of the Fermi-Dirac distribution function at 40 K. Different circle colors: different areas (Fig. S7). Colored lines: median (violet) surrounded by the standard deviation $\pm\sigma$ (green, blue). (c,d) Same as Fig. 3d, main text, but using a different reference voltage $V_{\text{ref}}$ to determine $R$. (c) $V_{\text{ref}} = 40\,\text{mV}$, (d) $V_{\text{ref}} = 60\,\text{mV}$.

anticorrelated with the gap size $\Delta$. This is demonstrated in Fig. S9b for $T = 40\,\mathrm{K}$. All $dI/dV(V)$ curves recorded at 4.3 K (area 1–3, 8000 curves) are convoluted with the derivative of the Fermi-Dirac distribution of 40 K (as in Fig. S9a, yellow curve) before $R$ is determined. Subsequently, each $R$ is related to its corresponding $\Delta$ (same $dI/dV(V)$ curve) as deduced via the method described in subsection S9 B. The anticorrelation of $R$ and $\Delta$ appears in Fig. S9b and is similarly found for all $T = 25 - 50\,\mathrm{K}$ (not shown). We used the resulting median of the simulated $R$ values (violet line in Fig. S9b) to deduce $\Delta$ from a measured $R$ for each $dI/dV(V)$ curve recorded at elevated $T$. The required simulated $R(T)$ curves for constant $\Delta$ are displayed as full lines in Fig. 3d, main text, for the sake of comparison with the $R$ values of the measured $\mathrm{d}I/\mathrm{d}V$ curves. The error of $R(\Delta)$ at given $T$ is taken as the $2\sigma$ width of the simulated $R(\Delta)$, marked by colored lines in Fig. S9b. For gap sizes below 10 meV, one observes an error of 2–4 meV only. Overall, the $R(\Delta)$ relation reaches a relative accuracy for $\Delta$ determination of $\pm 30\,\%$, as long as $\Delta$ remains below 20 meV. This error is displayed exemplarily on the very right of Fig. 3d, main text.

In addition, we determined errors for the measured $R$ values. They are deduced as the standard deviation of subsequently recorded 25 $\mathrm{d}I/\mathrm{d}V(V)$ data sets (averaged from 10 subsequent $\mathrm{d}I/\mathrm{d}V$ curves each and smoothed by box averaging of width 3 mV). The measurement errors are displayed as error bars at the data points in Fig. 3d, main text (circles). Interestingly, these measurement errors increase significantly around $T \simeq 45\,\mathrm{K}$, i.e., close to $T_\mathrm{C}$, where they get as large as 10 meV. Since the spatial drift during recording of the 25 data sets is below 1 nm, as deduced by comparing the long term development of $\mathrm{d}I/\mathrm{d}V$ data during cooling to the spatial fluctuations recorded at 4.3 K, these relatively large errors cannot be caused by a lateral drift of the tip with respect to the sample only. Likely, they are caused by enhanced temporal fluctuations of the gap close to $T_\mathrm{C}$ during recording of the 25 data sets. Thus, the increased error bars around $T_\mathrm{C}$ corroborate the relation of the band gap evolution to the magnetic properties additionally.

Error bars of the spatially averaged data points in Fig. 3d, main text, indicate the width of the $R$ histograms obtained at the corresponding $T$.

The reference voltage $V_\mathrm{ref} = -50\,\mathrm{mV}$ used to determine $R$ is chosen such that it is not influenced by temperature ($|eV_\mathrm{ref}| \gg 5k_\mathrm{B}T$) or gap size ($|eV_\mathrm{ref}| \gg \max(\Delta)/2$) and not influenced by spectroscopic features that might spatially vary due to disorder. We cross-checked that the exact value of $V_\mathrm{ref}$ barely influences the deduced $\Delta$. This is demonstrated

in Fig. S9c–d displaying the same data set as Fig. 3d, main text, but using two different $V_{\mathrm{ref}}$.

## S10: ELECTRIC TRANSPORT MEASUREMENTS

Transport invstigations were performed in the van der Pauw geometry with applied magnetic fields ranging from -3 T to +3 T oriented parallel to the rhombohedral axis of the epilayers. A mini cryogen-free system was employed for the magnetotransport measurements at temperatures between 2 K and 300 K. The results are shown in Fig. S10, where the Hall and anomalous Hall effect as well as the magnetoresistance are shown as a function of applied field and temperature. Evidently, the anomalous Hall effect appears at temperatures below about 50 K where a pronounced hysteresis appears in the transport data at fields less than about 1 T. This perfectly agrees with the onset of ferromagnetism detected by SQUID and XMCD at temperatures of 45 – 50K. The carrier concentration in the films was determined from the Hall resistance above 1 T, i.e., well above the coercive field. The deduced hole concentrations of the $MnSb_2Te_4$ samples range from 1 to $3 \cdot 10^{20}\mathrm{cm}^{-3}$, similar to values reported in the literature [5, 6].

## S11: DENSITY FUNCTIONAL THEORY CALCULATIONS

### A: Details of the Calculations

The electronic and magnetic structures were calculated by two different methods based on density functional theory (DFT). Most of the bulk-type calculations (Figs. 4f–k, main text, Fig. S11, Fig. S12a,b) used a Green function method within the multiple scattering theory [7, 8]. To describe both localization and interaction of the Mn 3$d$ orbitals appropriately, Coulomb $U$ values of 3–5 eV were employed within a GGA+$U$ approach [9]. During these calculations, we confirmed that different topological phases of $MnSb_2Te_4$ arise, if one uses either GGA+$U$ or LDA+$U$, indicating that LDA+$U$ is not sufficient. To account for antiferromagnetic configurations of $MnSb_2Te_4$, a double unit cell consisting of two septuple layers was used such as for Fig. 4k, main text, and Fig. S11a and c. In these structures, the Mn atoms couple ferromagnetically within the Mn planes and antiferromagnetically between neighboring Mn layers in adajacent septuple layers. For the determination of $T_{\mathrm{N}}$ and

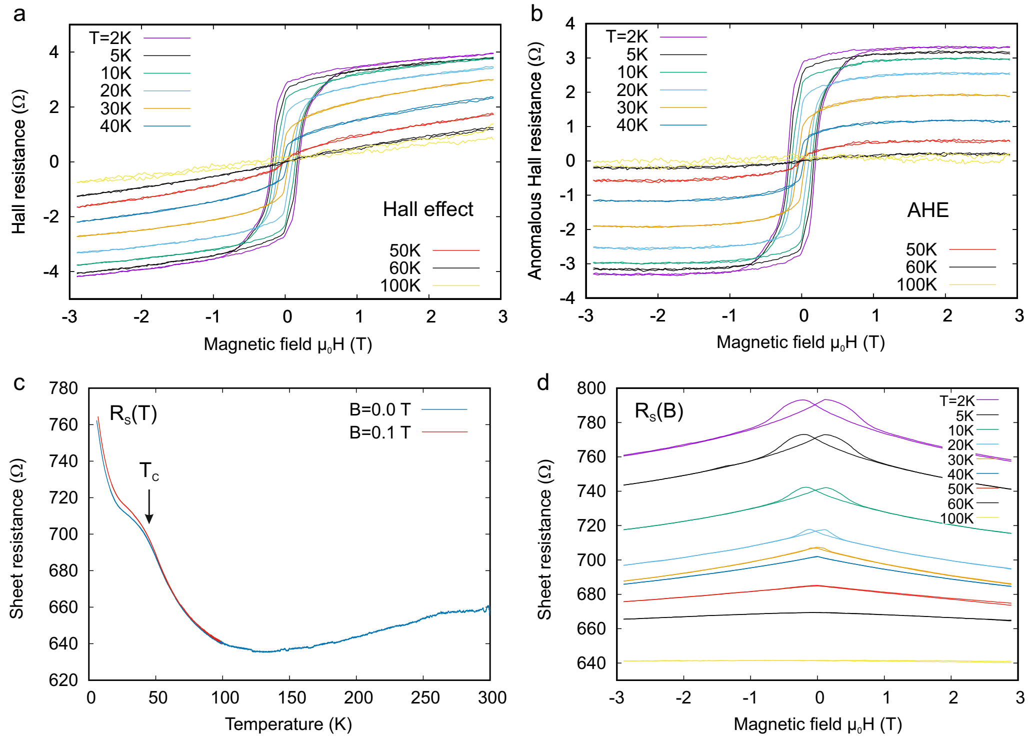


Figure S10. **Electrical transport of ferromangetic $MnSb_2Te_4$ films.** (a) Hall resistance, (b) anomalous Hall resistance and (d) magnetoresistance as a function of applied magnetic field recorded at various temperatures between 2 and 100 K, as indicated. Also shown in (c) is the sheet resistance as a function of temperature. All measurements clearly indicate ferromagnetic behavior with a $T_C$ close to 50 K, in agreement with SQUID and XMCD measurements.

$T_C$, exchange constants $J_{ij}$ were obtained by mapping the DFT calculations onto a classical Heisenberg model [10].

Different types of disorder were treated within a coherent potential approximation (CPA) [11, 12]. Chemical disorder is modelled by mixing various atomic species on the same atomic site (substitutional alloys). The elemental unit cell was used in this case such that only ferromagnetic order could be described. We simulated three types of chemical disorder, namely site exchange, i.e., placing as much Mn on Sb sites as Sb on Mn sites, Sb excess, i.e., additional, substitutional Sb in the Mn layers, and Mn excess, i.e., additional, substitutional Mn in the Sb layers. Electron (hole) doping was also modeled by CPA via mixing Te (Sb)

vacancies, that were simulated as empty spheres, with Te (Sb) atoms on the same site. Magnetic moment disorder was modeled by CPA via mixing two Mn atoms with opposite magnetic moment on the same atomic site. This approach has been proven to be very successful in mimicking spin moment fluctuations, e.g., to account for elevated temperatures. The 50 : 50 mixing represents the paramagnetic state, while smaller spin fluctuations are described by, e.g., 95 : 5 or 98 : 2 mixings.

The calculations in Fig. S12c–f were performed with the full-potential linearized augmented plane-wave method as implemented in the FLEUR code. Also here, GGA [13] with a Hubbard $U$ correction using $U = 6$ eV and $J = 0.54$ eV was used and spin-orbit coupling was included self-consistently in the non-collinear calculations [14]. Thin film calculations were also performed with the FLEUR code (Fig. 4c, main text, and Fig. S13b–e). These calculations are restricted to relatively small and simple unit cells in order to retain high enough accuracy to derive the Dirac cone dispersion and the size of its magnetic gap by DFT. Instead, the surface band structure for the antiferromagnetic ground state in Fig. 4d–e, main text, for the ferromagnetic ground state in Fig. 4b, main text, and for the mixed state in Fig. S13a was calculated by the projector augmented-wave method [15] using the VASP code [16, 17]. The exchange-correlation energy was treated using the GGA [13]. The Hamiltonian contained scalar relativistic corrections and the spin-orbit coupling was taken into account by the second variation method [18]. In order to describe the van der Waals interactions, we made use of the DFT-D3 [19, 20] approach. The Mn 3$d$ states were treated employing the GGA+$U$ approximation [9] within the Dudarev scheme [21]. The $U_{\text{eff}} = U - J$ value for the Mn 3$d$ states was chosen to 5.34 eV as in previous works [22–25].

For all three methods, the crystal structure of ideal $MnSb_2Te_4$ was fully optimized to obtain the equilibrium lattice parameters, namely cell volume, $c/a$ ratio as well as atomic positions. Using this structure yields the antiferromagnetic topological insulator state for $MnSb_2Te_4$ within both the projector augmented-wave and full-potential linearized augmented plane-wave methods (VASP and FLEUR, respectively). For the Green function method both the experimental crystal structure as determined by XRD (Fig. S2) and the theoretically optimized one result in the antiferromagnetic topological insulator phase.

In order to visualize the topological character within bulk-type band structure calculations, we analyze the spectral function difference between anion and cation contributions of each state: $A_k^i(E) = A_k^{\text{anion}}(E) - A_k^{\text{cation}}(E)$. The resulting $A_k^i(E)$ are displayed as color

| | | MnSb$_2$Te$_4$ | | | MnSb$_2$Te$_4$ with lattice | |
|---|---|---|---|---|---|---|
| | | 0% disorder | 5% disorder | 5% extra Mn | constants of MnBi$_2$Te$_4$ | |
| | | $T_N$=18 K | $T_C$=25 K | $T_C$=44 K | | $T_N$=20 K |
| direction | r (Å) | J (meV) | J (meV) | J (meV) | r (Å) | J (meV) |
| \|\| | 4.23 | 0.519 | 0.458 | 0.674 | 4.34 | 0.180 |
| \|\| | 7.36 | -0.024 | 0.034 | 0.024 | 7.51 | -0.007 |
| \|\| | 8.52 | 0.016 | 0.025 | 0.033 | 8.68 | 0.025 |
| ⊥ | 16.8 | -0.02 | 0.002 | 0.04 | 16.4 | -0.035 |

Table III. **Magnetic structure, critical temperature, and Mn exchange integrals of MnSb$_2$Te$_4$ for four different crystal configurations (DFT).** The exchange integrals $J$ for the four smallest distances $r$ between the contributing Mn ions are additionally marked by $\parallel$ ($\perp$) for intralayer (interlayer) Mn pairs. The model with 0% disorder refers to ideal MnSb$_2$Te$_4$ with all Mn atoms in the central plane of the septuple layers at the experimental lattice constants $a = 4.23$ Å, $c = 40.98$ Å (XRD). In the second model (5% disorder), a Mn-Sb site exchange leads to an occupancy of 95% Mn and 5% Sb in the central lattice plane and 2.5% Mn and 97.5% Sb in each of the two outer cationic lattice planes of the septuple layer. In the third case, 2.5% extra Mn was substitutionally introduced in the Sb layers, such that the outer cationic planes contain 2.5% Mn and 97.5% Sb, while a 100% Mn occupancy appears in the central layer. The fourth model features ideal MnSb$_2$Te$_4$ with 0% disorder, but laterally expanded to the lattice constants of MnBi$_2$Te$_4$, i.e., $a = 4.34$ Å, $c = 40.89$ Å.

code in Fig. 4f–k, main text, Fig. S11, and Fig. S12a,b with red color for $A^i_k(E) > 0$ and blue color for $A^i_k(E) < 0$. Band inversion can, hence, be deduced from a mutually changing color within adjacent bands.

### B: Magnetic Ground State for Different Disorder Configurations

Table III summarizes the magnetic properties for four different crystallographic configurations as obtained by bulk-type DFT calculations. The ideal MnSb$_2$Te$_4$ septuple layer configuration is antiferromagnetic with low $T_N = 18\,$K (third row). To probe the role of the lattice constant for the magnetic properties, we slightly expanded the lattice within the

planes towards the lattice constant of $MnBi_2Te_4$ (last row). This led to a reduced nearest neighbor exchange constant $J$, but barely to a change in $T_N$. Introducing 5 % Mn-Sb site exchange, in line with the XRD and STM data, turned the interlayer coupling ferromagnetic, but still with a $T_C$= 25 K only, i.e., significantly lower than the experimental value (fourth row). However, adding 2.5 % Mn to each Sb layer in the septuple without removing Mn from the central layer of the septuple, i.e., incorporation of 5 % excess Mn, yields $T_C$= 44 K, very close to the experimental value (fifth row). Hence, we conclude, in line with RBS, STM and XRD data, that an excess Mn in combination with an Sb deficiency is responsible for the high ferromagnetic Curie temperature via Mn substitution in the Sb layers.

### C: Topological Properties for Different Disorder Configurations

Figure S11 shows bulk band structure calculations for antiferromagnetic $MnSb_2Te_4$ with ideal stoichiometric order (a,c), ideal, ferromagnetic $MnSb_2Te_4$ (e) and ferromagnetic $MnSb_2Te_4$ with increasing Mn-Sb site exchange (b,d,f). Antiferromagnetic $MnSb_2Te_4$ is a topological insulator with inverted band gap at Γ, if spin-orbit coupling is considered (c). This is contrary to previous calculations that have suggested antiferromagnetic $MnSb_2Te_4$ to be trivial [26–29]. We assume that the topological insulator state of antiferromagnetic $MnSb_2Te_4$ was missed because structural optimization was either not performed in favor of experimental lattice constants [27, 29] or performed without van der Waals forces [26, 28] which both gave by $\sim 3\%$ larger $c$ parameters than in the structurally optimized equilibrium lattice. Ferromagnetic $MnSb_2Te_4$ instead, is a Weyl semimetal without band gap that remains a Weyl semimetal for moderate Mn-Sb site exchange (Fig. S11b,d), but becomes topologically trivial at large site exchange (Fig. S11f). Hence, site exchange alone does not reveal the experimentally observed ferromagnetic topological insulator in contrast to the magnetic disorder as presented in Fig. 4g–j, main text.

Figure S12 corroborates the gap opening by magnetic disorder. Figure S12b displays the complete band structure at maximum spin mixture (50 % spin-up, 50 % spin down) for each Mn lattice site as partially presented in Fig. 4j, main text. An inverted band gap of about 100 meV is found at Γ. Note that the purely ferromagnetic phase in Fig. S12a is calculated for a unit cell of a single septuple layer only and, hence, differs from Fig. S11e employing a unit cell with two septuple layers, due to backfolding.

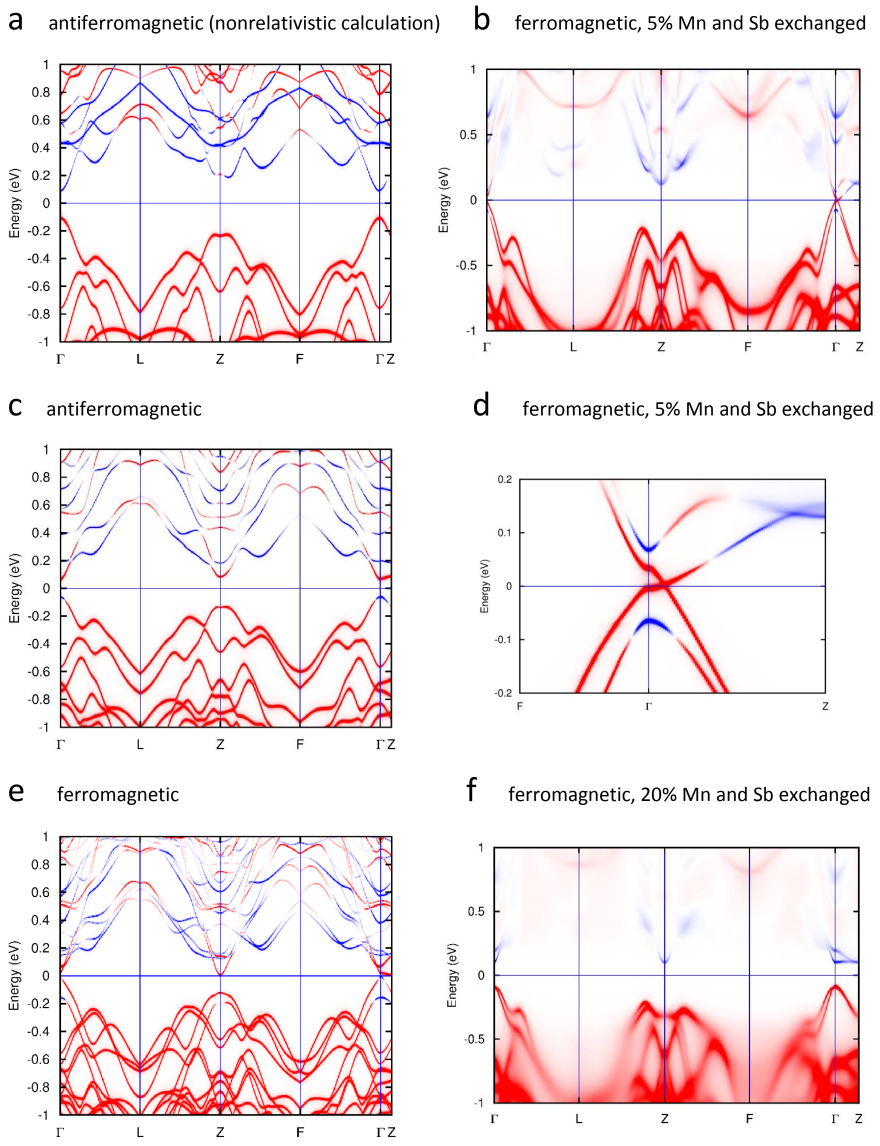


Figure S11. **Band Structure at Different Strength of Mn-Sb Site Exchange (DFT)**. Color represents the difference between anionic and cationic spectral function for each state, red: more anionic, blue: more cationic. (a) Antiferromagnetic, ideal $MnSb_2Te_4$ in a nonrelativistic (more precisely scalar relativistic) calculation. No band inversion occurs. (c) Antiferromagnetic, ideal $MnSb_2Te_4$ in a fully relativistic calculation yielding a band inversion. Only one inversion appears in the Brillouin zone (at Γ) evidencing a $Z_2$ topological insulator. (Also shown as Fig. 4k, main text, for the ferromagnetic unit cell.). (e) Same as (c) but for ferromagnetically ordered, ideal $MnSb_2Te_4$ with perfect out-of-plane alignment of Mn moments. A 3D Weyl semimetal occurs. The calculation used the antiferromagnetic unit cell for better comparison with (c). (b, d, f) Ferromagnetic $MnSb_2Te_4$ with different Mn-Sb site exchange as labeled (ferromagnetic unit cell). In (b) and (d), the Weyl point is preserved, while (f) shows a topologically trivial band gap.

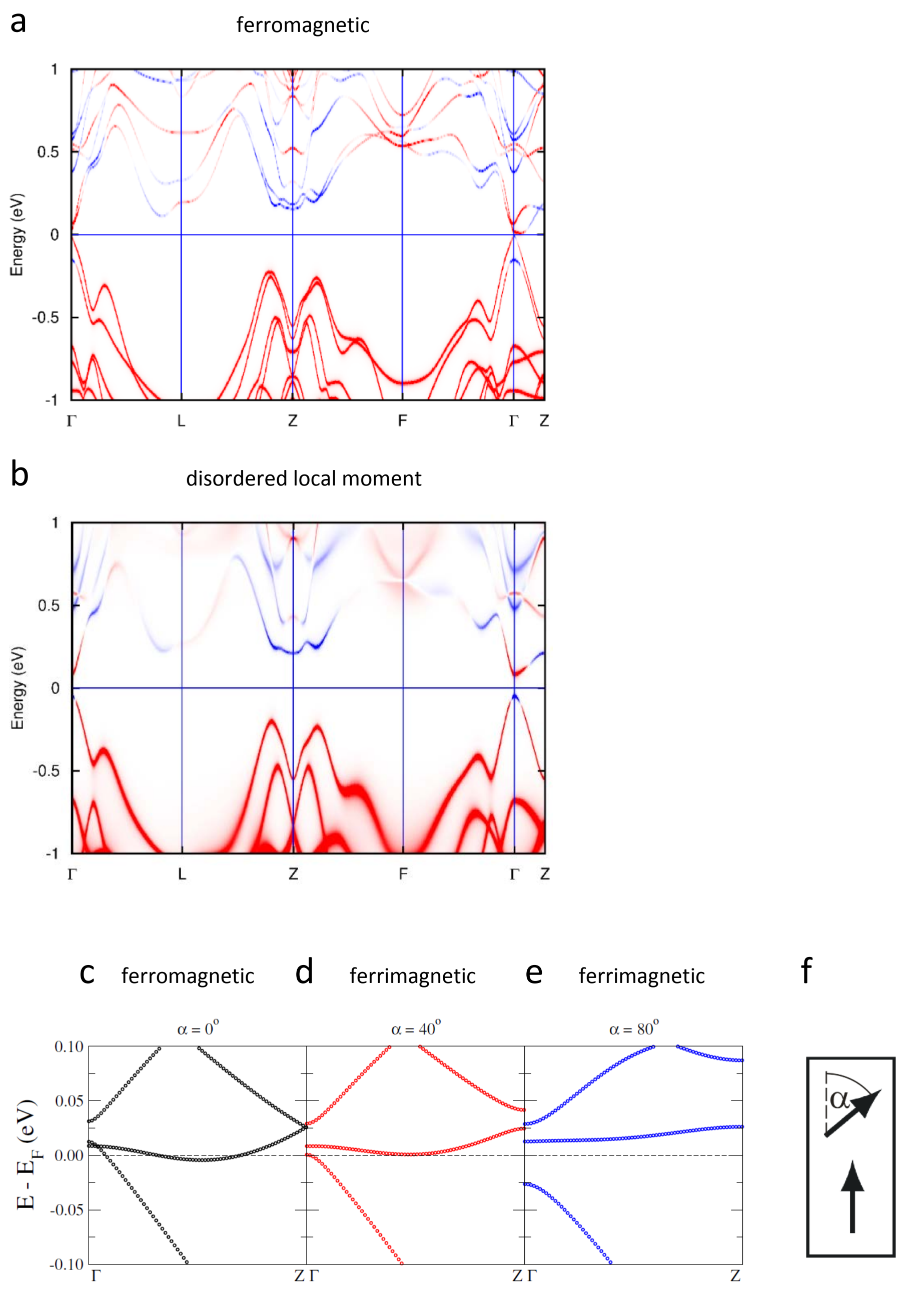


Figure S12. **Inverted band gap by magnetic disorder (DFT).** (a) Bulk band structure of ideal, ferromagnetic $MnSb_2Te_4$ with Mn moments perpendicular to the septuple layers (single layer unit cell). (Part of this figure is shown as Fig. 4f, main text.) (b) Same as (a), but with disordered local magnetic moments (50% spin up, 50% spin down). An inverted band gap appears at Γ. (Part of this figure is shown as Fig. 4j, main text.) (c–e) Alternative demonstration of gap opening at the Weyl point (two septuple layer unit cell): one layer exhibits a collinear out-of-plane ferromagnetic order, while the other is also collinear, but canted relative to the first one by an angle $\alpha$ as marked. (f) Vector model of the collinearly canted two septuple layers.

The Weyl point observed for the ferromagnetic $MnSb_2Te_4$ can also be opened by rotating the spins of adjacent Mn layers, while keeping a collinear spin order within each layer (Fig. S12c–f). Thus, magnetic disorder renders a dominantly ferromagnetic $MnSb_2Te_4$ a topological insulator as already discussed for Fig. 4g–j, main text.

Figure S13 shows the band structures of slab calculations for different magnetic disorder configurations, such that surface states are captured. They are performed, e.g., for a combination of ferromagnetic interior layers surrounded by a few antiferromagnetic layers on top and bottom (Fig. S13a). This configuration reveals a Dirac-type surface state with a gap around the Dirac point of 16 meV, very close to the average gap size observed by STS. The opposite configuration with antiferromagnetic interior surrounded by ferromagnetic surfaces also exhibits a gapped Dirac cone, here with 40 meV gap size, that might be enhanced by the thickness of this slab of only 7 septuple layers (Fig. S13d). Indeed, the spin-polarized states at the gap edge penetrate about 3 septuple layers into the bulk of the thin film (Fig. S13b). The out-of-plane spin polarization near the Dirac point amounts to $\sim 60\%$, nicely matching the experimentally found out-of-plane spin polarization in spin-resolved ARPES (Fig. 2j, main text). The latter amounts to $\sim 25\,\%$ at 30 K in line with the reduced magnetization at this elevated $T$ (Fig. 1d,f, main text). Note that the band structure in Fig. S13d also features an exchange splitting of bulk bands as visible by the different colors around $-0.2$ eV.

The pure antiferromagnetic configuration (Fig. S13e) shows a small band gap of the topological surface state as well, while the pure ferromagnetic order leads to a gapped Weyl cone (Fig. S13c), likely being an artifact of the finite slab size of 7 septuple layers only.

**D: Influence of Charge Doping on the Magnetic Interactions**

Finally, the influence of charge doping on the magnetic properties of $MnSb_2Te_4$ was studied. For undoped, ideally stacked $MnSb_2Te_4$, the leading magnetic interaction between the septuple layers is of a superexchange type, which is responsible for the antiferromagnetic interlayer coupling. As shown above, chemical disorder by exchanging (replacing) Mn with (by) Sb changes the magnetic order to ferromagnetic. The presence of Mn defects could induce an additional Ruderman-Kittel-Kasuya-Yoshida (RKKY) interaction that might influence the magnetic order, if mobile charges are present. However, our calculations show only minor changes of exchange constants and the magnetic transition temperatures upon

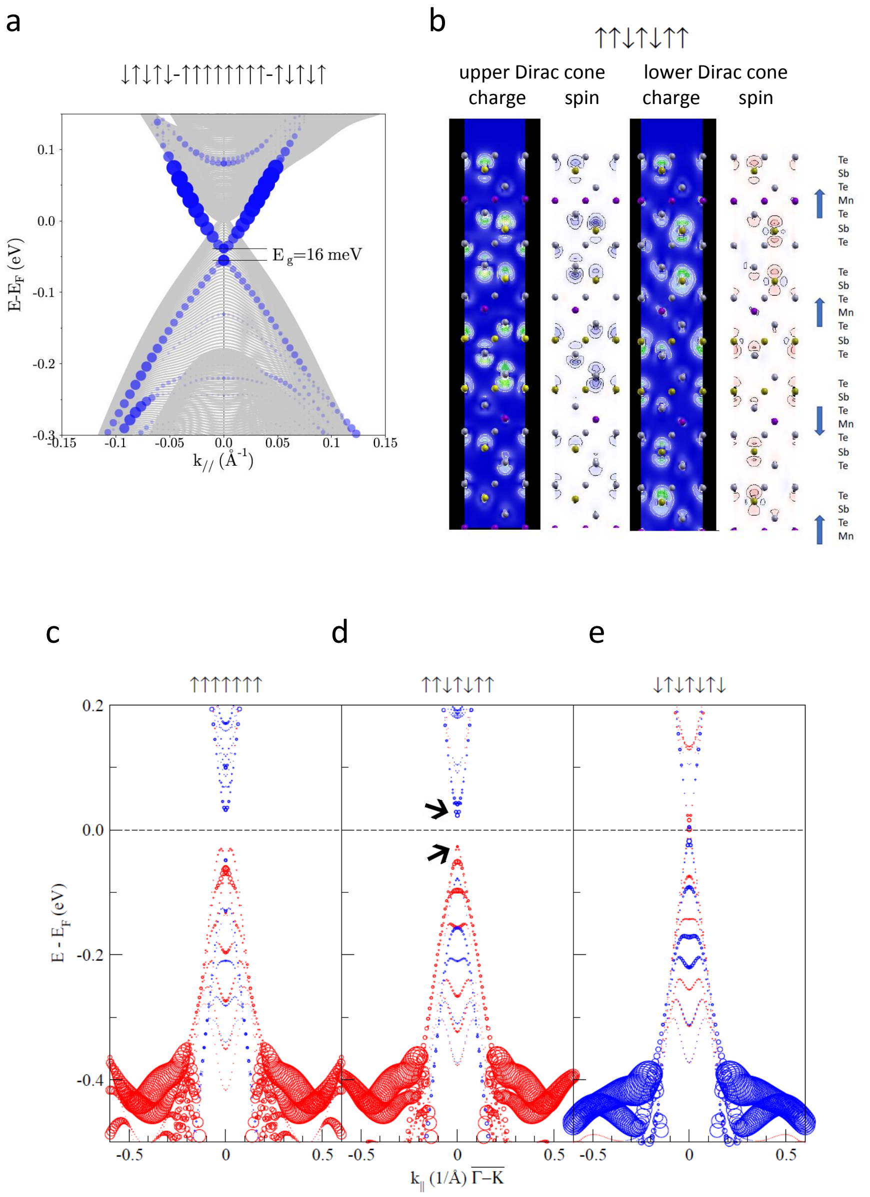


Figure S13. **Influence of magnetic structure on topological surface states (DFT).** (a) Band structure for a ferromagnetic bulk surrounded by antiferromagnetic surface layers as marked on top. Blue dots are surface states with dot diameter marking their strength at the surface septuple layer. A gap appears in the topological surface state. (b) Charge density and spin density of the states at the edges of the magnetically induced band gap in (d) displayed as thin film cross section. The magnetic configuration is marked on top. (c–e) Band structure for various magnetic configurations each marked on top. Colors denote the spin density in the out-of-plane direction. (c) Ferromagnetic order with out-of-plane anisotropy. (d) Two ferromagnetic septuple layers on both sides of three antiferromagnetic layers. Black arrows mark the states shown in (b). (e) Perfect antiferromagnetic order as in Fig. 4d,e, main text.

charge doping. For n-type doping by 0.2% Te vacancies, $E_{\rm F}$ shifts by 0.29 eV increasing $T_{\rm C}$ by 0.9 K only. For p-type doping by 0.2% Sb vacancies, $E_{\rm F}$ shifts by $-0.08$ eV increasing $T_{\rm C}$ by 1.6 K. This indicates that the RKKY interaction is negligible in $MnSb_2Te_4$.

[1] M. Mayer, *Nuclear Instrum. Methods B* **2014**, *332* 176.

[2] H. Steiner, V. Volobuev, O. Caha, G. Bauer, G. Springholz, V. Holý, *J. Appl. Cryst.* **2014**, *47*, 6 1889.

[3] E. D. L. Rienks, S. Wimmer, J. Sánchez-Barriga, O. Caha, P. S. Mandal, J. Růžička, A. Ney, H. Steiner, V. V. Volobuev, H. Groiss, M. Albu, G. Kothleitner, J. Michalička, S. A. Khan, J. Minár, H. Ebert, G. Bauer, F. Freyse, A. Varykhalov, O. Rader, G. Springholz, *Nature* **2019**, *576*, 7787 423.

[4] M. Morgenstern, *Surf. Rev. Lett.* **2003**, *10*, 06 933.

[5] J.-Q. Yan, S. Okamoto, M. A. McGuire, A. F. May, R. J. McQueeney, B. C. Sales, *Phys. Rev. B* **2019**, *100*, 10 104409.

[6] Y. Chen, Y.-W. Chuang, S. H. Lee, Y. Zhu, K. Honz, Y. Guan, Y. Wang, K. Wang, Z. Mao, J. Zhu, C. Heikes, P. Quarterman, P. Zajdel, J. A. Borchers, W. Ratcliff, *Phys. Rev. Mat.* **2020**, *4*, 6 064411.

[7] B. L. Gyorffy, M. J. Stott, In D. J. Fabian, L. M. Watson, editors, *Proc. of the Int. Conf. on Band Structure and Spectroscopy of Metals and Alloys*. Academic Press, **1973** 385.

[8] M. Geilhufe, S. Achilles, M. A. Köbis, M. Arnold, I. Mertig, W. Hergert, A. Ernst, *J. Phys.: Condens. Matter* **2015**, *27*, 43 435202.

[9] V. I. Anisimov, J. Zaanen, O. K. Andersen, *Phys. Rev. B* **1991**, *44*, 3 943.

[10] A. Liechtenstein, M. Katsnelson, V. Antropov, V. Gubanov, *J. Magn. Magn. Mat.* **1987**, *67*, 1 65.

[11] P. Soven, *Phys. Rev.* **1967**, *156*, 3 809.

[12] B. L. Gyorffy, *Phys. Rev. B* **1972**, *5*, 6 2382.

[13] J. P. Perdew, K. Burke, M. Ernzerhof, *Phys. Rev. Lett.* **1996**, *77*, 18 3865.

[14] P. Kurz, F. Förster, L. Nordström, G. Bihlmayer, S. Blügel, *Phys. Rev. B* **2004**, *69* 024415.

[15] P. E. Blöchl, *Phys. Rev. B* **1994**, *50*, 24 17953.

[16] G. Kresse, J. Furthmüller, *Phys. Rev. B* **1996**, *54*, 16 11169.

[17] G. Kresse, D. Joubert, *Phys. Rev. B* **1999**, *59*, 3 1758.

[18] D. D. Koelling, B. N. Harmon, *Journal of Physics C: Solid State Physics* **1977**, *10*, 16 3107.

[19] S. Grimme, J. Antony, S. Ehrlich, H. Krieg, *J. Chem. Phys.* **2010**, *132*, 15 154104.

[20] S. Grimme, S. Ehrlich, L. Goerigk, *J. Comput. Chem.* **2011**, *32*, 7 1456.

[21] S. L. Dudarev, G. A. Botton, S. Y. Savrasov, C. J. Humphreys, A. P. Sutton, *Phys. Rev. B* **1998**, *57*, 3 1505.

[22] S. Eremeev, M. Otrokov, E. Chulkov, *J. Alloys Compd.* **2017**, *709* 172.

[23] M. M. Otrokov, T. V. Menshchikova, M. G. Vergniory, I. P. Rusinov, A. Y. Vyazovskaya, Y. M. Koroteev, G. Bihlmayer, A. Ernst, P. M. Echenique, A. Arnau, E. V. Chulkov, *2D Mater.* **2017**, *4*, 2 025082.

[24] M. M. Otrokov, I. I. Klimovskikh, H. Bentmann, D. Estyunin, A. Zeugner, Z. S. Aliev, S. Gaß, A. U. B. Wolter, A. V. Koroleva, A. M. Shikin, M. Blanco-Rey, M. Hoffmann, I. P. Rusinov, A. Y. Vyazovskaya, S. V. Eremeev, Y. M. Koroteev, V. M. Kuznetsov, F. Freyse, J. Sánchez-Barriga, I. R. Amiraslanov, M. B. Babanly, N. T. Mamedov, N. A. Abdullayev, V. N. Zverev, A. Alfonsov, V. Kataev, B. Büchner, E. F. Schwier, S. Kumar, A. Kimura, L. Petaccia, G. D. Santo, R. C. Vidal, S. Schatz, K. Kißner, M. Ünzelmann, C. H. Min, S. Moser, T. R. F. Peixoto, F. Reinert, A. Ernst, P. M. Echenique, A. Isaeva, E. V. Chulkov, *Nature* **2019**, *576*, 7787 416.

[25] M. Otrokov, I. Rusinov, M. Blanco-Rey, M. Hoffmann, A. Vyazovskaya, S. Eremeev, A. Ernst, P. Echenique, A. Arnau, E. Chulkov, *Phys. Rev. Lett.* **2019**, *122*, 10 107202.

[26] D. Zhang, M. Shi, T. Zhu, D. Xing, H. Zhang, J. Wang, *Phys. Rev. Lett.* **2019**, *122*, 20 206401.

[27] B. Chen, F. Fei, D. Zhang, B. Zhang, W. Liu, S. Zhang, P. Wang, B. Wei, Y. Zhang, Z. Zuo, J. Guo, Q. Liu, Z. Wang, X. Wu, J. Zong, X. Xie, W. Chen, Z. Sun, S. Wang, Y. Zhang, M. Zhang, X. Wang, F. Song, H. Zhang, D. Shen, B. Wang, *Nat. Commun.* **2019**, *10*, 1 4469.

[28] C. Lei, S. Chen, A. H. MacDonald, *Proc. Natl. Acad. Sci.* **2020**, *117*, 44 27224.

[29] Y. Liu, L.-L. Wang, Q. Zheng, Z. Huang, X. Wang, M. Chi, Y. Wu, B. C. Chakoumakos, M. A. McGuire, B. C. Sales, W. Wu, J. Yan, *Phys. Rev. X* **2021**, *11* 021033.
